# Comprehensive Data Integration Approach to Assess Immune Responses and Correlates of RTS,S/AS01-Mediated Protection From Malaria Infection in Controlled Human Malaria Infection Trials

**DOI:** 10.3389/fdata.2021.672460

**Published:** 2021-06-15

**Authors:** William Chad Young, Lindsay N. Carpp, Sidhartha Chaudhury, Jason A. Regules, Elke S. Bergmann-Leitner, Christian Ockenhouse, Ulrike Wille-Reece, Allan C. deCamp, Ellis Hughes, Celia Mahoney, Suresh Pallikkuth, Savita Pahwa, S. Moses Dennison, Sarah V. Mudrak, S. Munir Alam, Kelly E. Seaton, Rachel L. Spreng, Jon Fallon, Ashlin Michell, Fernando Ulloa-Montoya, Margherita Coccia, Erik Jongert, Galit Alter, Georgia D. Tomaras, Raphael Gottardo

**Affiliations:** ^1^ Vaccine and Infectious Disease Division, Fred Hutchinson Cancer Research Center, Seattle, WA, United States; ^2^ Malaria Biologics Branch, Walter Reed Army Institute of Research, Silver Spring, MD, United States; ^3^ PATH Malaria Vaccine Initiative, Washington, DC, United States; ^4^ Department of Microbiology and Immunology, University of Miami Miller School of Medicine, Miami, FL, United States; ^5^ Center for Human Systems Immunology, Duke University, Durham, NC, United States; ^6^ Departments of Surgery, Immunology, and Molecular Genetics and Microbiology, Duke University, Durham, NC, United States; ^7^ Duke Human Vaccine Institute, Duke University, Durham, NC, United States; ^8^ Department of Pathology, Duke University, Durham, NC, United States; ^9^ Ragon Institute of MGH, MIT, and Harvard, Cambridge, MA, United States; ^10^ GSK, Rixensart, Belgium

**Keywords:** correlates of protection, immune response, malaria, vaccine, machine learning

## Abstract

RTS,S/AS01 (GSK) is the world’s first malaria vaccine. However, despite initial efficacy of almost 70% over the first 6 months of follow-up, efficacy waned over time. A deeper understanding of the immune features that contribute to RTS,S/AS01-mediated protection could be beneficial for further vaccine development. In two recent controlled human malaria infection (CHMI) trials of the RTS,S/AS01 vaccine in malaria-naïve adults, MAL068 and MAL071, vaccine efficacy against patent parasitemia ranged from 44% to 87% across studies and arms (each study included a standard RTS,S/AS01 arm with three vaccine doses delivered in four-week-intervals, as well as an alternative arm with a modified version of this regimen). In each trial, RTS,S/AS01 immunogenicity was interrogated using a broad range of immunological assays, assessing cellular and humoral immune parameters as well as gene expression. Here, we used a predictive modeling framework to identify immune biomarkers measured at day-of-challenge that could predict sterile protection against malaria infection. Using cross-validation on MAL068 data (either the standard RTS,S/AS01 arm alone, or across both the standard RTS,S/AS01 arm and the alternative arm), top-performing univariate models identified variables related to Fc effector functions and titer of antibodies that bind to the central repeat region (NANP6) of CSP as the most predictive variables; all NANP6-related variables consistently associated with protection. In cross-study prediction analyses of MAL071 outcomes (the standard RTS,S/AS01 arm), top-performing univariate models again identified variables related to Fc effector functions of NANP6-targeting antibodies as highly predictive. We found little benefit–with this dataset–in terms of improved prediction accuracy in bivariate models vs. univariate models. These findings await validation in children living in malaria-endemic regions, and in vaccinees administered a fourth RTS,S/AS01 dose. Our findings support a “quality as well as quantity” hypothesis for RTS,S/AS01-elicited antibodies against NANP6, implying that malaria vaccine clinical trials should assess both titer and Fc effector functions of anti-NANP6 antibodies.

## Introduction

Malaria remains a leading cause of global morbidity and mortality, and disproportionately affects children <5 years old in the World Health Organization (WHO) African Region (World Health Organization, 2020). The causative agents of malaria, *Plasmodium* parasites (especially *P. falciparum* and *P. vivax*), are transmitted to humans through the bite of infected female *Anopheles* mosquitoes (World Health Organization, 2020). Although some effective prevention tools (e.g., long-lasting insecticidal nets) have been developed, a durably efficacious preventive vaccine will be required to meet the challenge of eradicating malaria (Wilson et al., 2019).

The RTS,S/AS01 vaccine (GSK) is the most clinically advanced vaccine to date (Laurens, 2020) and targets the pre-erythrocytic stage of *P. falciparum* by eliciting antibody and T-cell responses to circumsporozoite protein (CSP), the major surface protein of sporozoites (Hoffman et al., 2015). In a phase 3 trial in African infants and children, estimated vaccine efficacy (VE) of RTS,S/AS01E against clinical malaria over a follow-up time of 12 months post-final dose was 56% in young children (5–17 months old) and 31% in infants (6–12 weeks old) (RTS, S Clinical Trials Partnership, 2011; RTS, S Clinical Trials Partnership, 2012; RTS, S Clinical Trials Partnership, 2014; RTS, S Clinical Trials Partnership, 2015). RTS,S/AS01 obtained a positive scientific opinion from the European Medicines Agency in 2015 (Hawkes, 2015) and was recommended by the WHO for a pilot implementation program in Ghana, Malawi, and Kenya that initiated in 2019 (World Health Organization, 2019).

**FIGURE 1:**
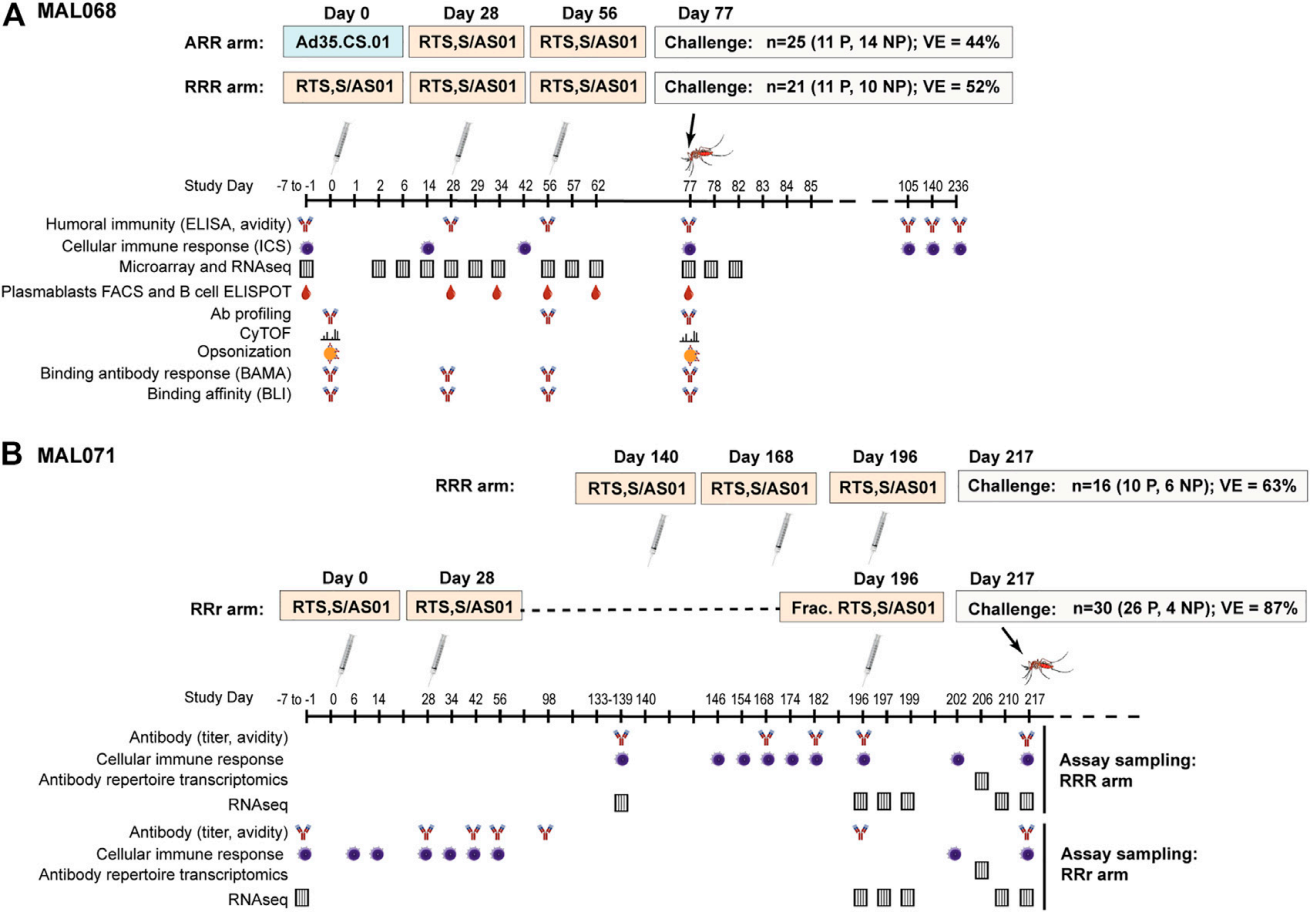
Schemas, efficacy results, and sampling schedules for the MAL068 and MAL071 studies. MAL071 study days past 217 (challenge day) are not shown, but follow-up continued until Day 376.

Controlled human malaria infection (CHMI) studies are a valuable tool for identifying correlates of protection, including in the context of vaccine development (Stanisic et al., 2018). MAL068 [NCT01366534 (Ockenhouse et al., 2015)] and MAL071 [NCT01857869 (Regules et al., 2016)] were randomized phase 2a studies (MAL068 observer blind, MAL071 open-label) designed to evaluate the safety, reactogenicity, immunogenicity, and efficacy of RTS,S/AS01 against sporozoite challenge of healthy, malaria-naïve adults. A schema of each study and a summary of the results are shown in Figure 1.

Each trial included a standard arm with three RTS,S/AS01 doses administered at one-month intervals (RRR arm), as well as an alternative arm with a modified version of this regimen (Figure 1). In MAL068, the alternative arm consisted of one dose of replication-incompetent, recombinant adenovirus 35 expressing circumsporozoite protein (Ad35.CS.01), followed by two doses of RTS,S/AS01 (ARR arm). In MAL071, the alternative arm was a delayed fractional-third-dose arm consisting of two doses of RTS,S/AS01 one month apart, and a one-fifth fractional dose 6 months later (RRr arm). At three weeks post-last vaccination, all participants were challenged with infected *P. falciparum* mosquitoes and monitored for parasitemia through Day 28 post-challenge. In the RRR arms, VE against malaria infection was 52% (95% CI 25–70%) in MAL068 and 63% (29–80%) in MAL071. In the alternative arms, VE against malaria infection was 44% (21–60%) in the ARR arm of MAL068 and 87% (67–95%) in the RRr arm of MAL071.

A large body of work (Ockenhouse et al., 2015; Chaudhury et al., 2016; Kazmin et al., 2017; Dennison et al., 2020; Du et al., 2020; Pallikkuth et al., 2020; Suscovich et al., 2020; Seaton et al. under revision)^1^ has been conducted using immune response data from MAL068 and/or MAL071 to identify correlates of RTS,S/AS01-mediated protection in malaria-naïve adults, yielding many insights and generating hypotheses about the mechanism of protection in this population. The immunodominant amino acid repeat region (NANP) of CSP is the main target of the B-cell response to CSP (White et al., 2015), and day-of-challenge anti-NANP repeat region IgG antibody titers have been shown to associate with protection (Ockenhouse et al., 2015). However, there does not appear to be a quantitative cut-off associated with protection (Ockenhouse et al., 2015; Suscovich et al., 2020), indicating that other immune responses also play a role as discussed further below.

Du et al. reported that the MX2/GPR183 expression ratio in peripheral blood mononuclear cells (PBMCs) at 1 day post-third dose discriminated protected from non-protected RTS,S/AS01 recipients in five independent CHMI trials, including MAL068 and MAL071 (Du et al., 2020). Specifically, participants with higher fold-change from baseline to 1 day after the third immunization were more likely to be protected. GPR183 (also known as EBI2), along with its ligand 7α,25-dihydroxycholesterol, plays an important role in T-cell dependent antibody responses by positioning different immune cell types at the appropriate compartments in secondary lymphoid organs such as the spleen (Barington et al., 2018). Upregulation of GPR183 helps localize activated CD4^+^ T cells, B cells, and dendritic cells to the T zone—B-cell follicle interface (Gatto and Brink, 2013; Li et al., 2016; Lu et al., 2017). In CD4^+^ T cells, GPR183 upregulation also augments T peripheral follicular helper cell (Tfh) cell development at early time points and is crucial for the generation of extrafollicular plasma cell responses (Gatto and Brink, 2013; Li et al., 2016). Relatedly, Pallikuth et al. showed that early (day 6 post-first dose) CSP-specific pTfh responses, along with durable CSP-specific memory B cell responses emerging post-dose two, were associated with protection in MAL071 (Pallikkuth et al., 2020). These findings are consistent with a model wherein localization of GPR183-expressing CD4^+^ T cells, B cells, and/or dendritic cells to the T zone—B-cell follicle interface (leading to a higher MX2/GPR183 ratio, as GPR183-expressing cells leave the peripheral compartment) is crucial for development of the protective CSP-specific pTfh and memory B cell responses seen by Pallikkuth et al. (2020).

Kazmin et al. reported an inverse correlation with protection of natural killer (NK) cell-related blood transcriptional module expression in PBMCs, in both arms of MAL068 (Kazmin et al., 2017), generating the hypothesis that vaccination stimulates migration of circulating NK cells to lymphoid tissues/organs such as the liver (i.e., leading to a reduction of NK cells in the peripheral blood) where the NK cells might contribute to the killing of infected cells through mechanisms such as antibody-dependent cellular cytotoxicity (ADCC).

Phagocytosis *via* opsonophagocytic pathways such as antibody-dependent cellular phagocytosis (ADCP) of parasites can have widely divergent outcomes [reviewed in Leitner et al. (2020)]. On the one hand, phagocytic cell uptake of opsonized parasites can result in parasite clearance by antibody-dependent cellular phagocytosis as well as immune-cell activation and secretion of inflammatory molecules. This outcome was mechanistically demonstrated in a recent “systems serology” analysis of >100 antibody features (Fc receptor binding, effector functions, etc.) of anti-CSP (full length), anti-NANP6, and anti-CSP C-terminal region antibodies in MAL068 vaccinees (Suscovich et al., 2020), which implicated both NANP6-specific ADCP and FcGRIIIA [expressed by natural killer (NK) cells (Vivier et al., 2008)] binding of NANP6-specific antibodies in protection (Suscovich et al., 2020). Similarly, opsonization by CSP-specific antibodies was proposed to contribute to RTS,S/AS01-induced protection (Schwenk et al., 2003).

On the other hand, parasites can also both evade and actively manipulate the host immune response *via* “phagosomal escape”, where phagocytosed parasites reside in the phagocytic immune cell and may also influence its function to undermine the host defense (Leitner et al., 2020). Chaudhury et al. reported that the “opsonization index” (corresponding to phagocytic activity normalized to anti-CSP antibody titer) was an inverse correlate of protection in MAL068 and that opsonophagocytic activity was likely mediated by C-terminal-specific antibodies (Chaudhury et al., 2016). A subsequent study by Chaudhury et al. presented evidence that, in MAL071, anti-CSP IgG4 antibodies inhibited opsonophagocytic activity of other anti-CSP IgG antibody subclasses. Subsequent analyses have shown that most opsonophagocytic antibodies are C-terminal-specific (rather than NANP6-specific) and that lower opsonophagocytic activity was associated with protection across all RTS,S/AS01 trials analyzed. The apparently conflicting findings regarding opsonophagocytic activity in RTS,S/AS01 vaccinees could be due to the different approaches taken: dividing CSP-targeted phagocytosis by CSP antigen titer (Chaudhury et al., 2016) vs. directly assessing CSP antigen-targeted phagocytosis (Suscovich et al., 2020).

Most recently, Seaton et al. examined a large number of CSP-specific antibody features (magnitude, subclass, and avidity) as humoral correlates of protection in the MAL068 and MAL071 trials, reporting that magnitude of NANP6-specific IgG1 antibody was the most predictive variable of protection in univariate models (Seaton et al., under revision)^1^.

This substantial body of MAL068/MAL071 correlates work has included antibody, cellular, and transcriptomic assays performed by many groups across different research organizations. Papers have focused on limited subsets of data and have not integrated all available immune response data across the two studies, nor have they used the same approach or mathematical tools for assessing a given immune response measurement as a correlate of protection (CoP).

Given that CHMI trials are resource-intensive to conduct, it is worthwhile to mine the few valuable datasets that are currently available, using a variety of approaches and techniques. For the first time, here we considered the entire repertoire of available MAL068 and MAL071 assay data in a correlates analysis, including all immune response variables in a unified data analysis procedure. We focused on day-of-challenge data because of the broad availability of data at this time-point, in order to highlight the integration aspect across assays. We evaluated the performance of different predictive computational models, including univariate and bivariate, in predicting individual-level outcomes in the MAL068 and the MAL071 trials. In this analysis, we identify NANP6-targeted antibody dependent cellular phagocytosis (ADCP) and anti-NANP6 IgG1 antibody titer as consistently predictive immune response measurements, with higher levels of each biomarker on day of challenge associated with individual-level protection after controlled malaria challenge. Our findings suggest that univariate models perform as well or better (in terms of predictive probability) than bi- or trivariate models, at least in this dataset, and provide the first head-to-head comparison (where the same pre-processing and statistical methodology has been used) between the previously published CoPs corresponding to day-of-challenge immune response measurements.

## Materials and Methods

### Available Assay Data

In MAL068 and MAL071, longitudinal PBMC and serum sampling was performed from enrollment through challenge, enabling assessment of a broad array of antibody, cellular, and transcriptomic immune responses that were included among the biomarkers analyzed in this study. Figure 1 denotes the studies assessed using either PBMC or serum samples and the corresponding sample timepoints in days from first vaccination. Table 1 provides additional information on the assay laboratories and references for prior publications describing the methods for sample processing, immunoassays, and data processing, if applicable. While the sampling schedules differed for the various immunoassays, day of challenge was the most common sampling day across assays (Figure 1) and hence only day-of-challenge data were considered in this integrative analysis.

**TABLE 1:** Available laboratory assay data from each trial. Bolded data are data used in this integration.

MAL068			MAL071		
Data type	Source lab	Reference	Data type	Source lab	Reference
**Gene expression**					
Microarray	Emory	Kazmin et al. (2017)			
**RNA-seq**	**CIDR**	**Du et al. (2020)**	**RNA-seq**	**CIDR, Emory**	**Du et al. (2020)**
**Cellular**					
**ICS**	**GSK**	**Ockenhouse et al. (2015); Kazmin et al. (2017)**	**ICS^e^**	**GSK, U Miami**	**Regules et al. (2016); Pallikkuth et al. (2020)**
**T-cell ELISpot**	**Crucell/Janssen**	**Ockenhouse et al. (2015)**	**Peripheral follicular helper cell (pTfh) frequency and function**	**U Miami**	**Pallikkuth et al. (2020)**
**Antibody**					
**Seroneutralization assay**	**Crucell/Janssen**	**Ockenhouse et al. (2015); Kazmin et al. (2017)**	**B-cell ELISpot**	**U Miami**	**Pallikkuth et al. (2020)**
Ab-mediated opsonization	WRAIR	Chaudhury et al. (2016)	Ab-mediated opsonization	WRAIR	Chaudhury et al. (2017)
**Ab functionality** ^b^	**MGH**	**Suscovich et al. (2020)**	**Ab functionality**	**MGH**	**Unpublished**
**ELISA^c^**	**CEVAC, GSK, WRAIR**	**Ockenhouse et al. (2015); Chaudhury et al. (2016); Radin et al. (2016)**	**ELISA–Titer^d^**	**CEVAC, GSK, WRAIR, U Miami**	**Radin et al. (2016); Regules et al. (2016); Chaudhury et al. (2017)**
			**ELISA–Avidity^e^**		
**BAMA IgG Subclass (IgG1-4)^f^ & BLI Ab Avidity^g^**	**Duke**	**Seaton et al. (under revision)^1^**	**BLI Ab Avidity^h^**	**Duke**	**Seaton et al. (under revision)^1^**
			**BAMA and BLI IgG Subclass (IgG 1–4)^i^**		
			**Luminex^j^**	**WRAIR, U Miami**	**Chaudhury et al. (2017); Pallikkuth et al. (2020); Pallikkuth et al. (2020)**
			B cell phenotyping	U Miami	
**Other**					
Microscopy		Ockenhouse et al. (2015)	Microscopy		Regules et al. (2016)
PCR		Ockenhouse t al. (2015)	PCR		Regules et al. (2016)

a Includes: T cells, peripheral T follicular helper (pTfh) cells, B cells.

b Includes: antibody-dependent cellular cytoxicity (ADCC), antibody-dependent complement deposition (ADCD), antibody-dependent cellular phagocytosis (ADCP), antibody dependent dendritic cell phagocytosis (ADDCP), antibody-dependent natural killer cell activation (ADNKA), antibody-dependent neutrophil phagocytosis (ADNP), Fc array, and Ig subclass.

c Includes: anti-circumsporozoite (CS), anti-Hepatitis B surface antigen (HBsAg), anti-full length CS and anti-NANP6 IgG titers; anti-CS IgG avidity.

d Includes: anti-CS, anti-full length CSP, anti-C-term, anti-NANP, and anti-HBsAg IgG titers.

e Includes: anti-full length CSP, anti-C-term, anti-NANP, anti-CS repeat region IgG avidity may be transferred at a later date.

f Includes: binding antibody multiplex assay (BAMA) full length circumsporozoite protein (CSP), NANP6, NPNA3, C-term, and Hepatitis B subclass (IgG1-4).

g Includes: biolayer interferometry (BLI) full length CSP, C-term, NANP6, NPNA3, and N-interface.

h Includes: full length CSP, NANP6, NPNA3, N-interface, C-term BLI Ab Avidity data.

i Includes: BAMA full length CSP IgG1-4, NANP6 IgG1-4, C-term IgG3, and Hepatitis B IgG3 and BLI full-length CSP subclass avidity.

j Includes: full-length CSP, C-term, NANP6 IgG1-4 subclass titer

### Lab Methods: Antigens, Monoclonal Antibodies, BAMA, and BLI Avidity Assay

#### Antigens and Monoclonal Antibodies

A recombinant CSP (CSP-FL) containing the N-terminal region, three NVDP and 19 NANP repeats followed by the C-terminal region was produced and purified as described previously (Schwenk et al., 2014). Synthetic peptides NANP6 and C-term corresponding to the central repeat and carboxy terminal regions of CSP respectively were made with an amino terminal biotin-Aminohexanoic acid (biotin-Ahx) tag. NANP6 (biotin-Ahx-NANPNANPNANPNANPNANPNANP) and the negative control peptide antigen C1 (Biotin-KKMQEDVISL WDQSLKPCVK LTPLCV) were obtained from CPC Scientific (Sunnyvale, CA). The C-term PF16 antigen (biotin-Ahx- EPSDKHIKEY LNKIQNSLST EWSPCSVTCG NGIQVRIKPG SANKPKDELD YANDIEKKIC KMEKCS with an amidated carboxy terminal) was procured from Biomatik (Cambridge, ON, Canada). NPNA3 (biotin-Ahx-NPNANPNANPNA with an amidated carboxy terminal) and N-interface (biotin-Ahx-KQPADGNPDPNANPN with an amidated carboxy terminal) were custom made by CPC Scientific. Vaccine-matched Hepatitis B (HepB) antigen was obtained from GSK. The negative control used in BLI assays, Ovalbumin-biotin, was purchased from Galab Laboratories (Hamburg, Germany). Previously described (Dennison et al., 2018) recombinant monoclonal antibodies (mAbs) AB334 and AB236 that are specific for the central repeat and C-terminal regions of CSP, respectively, were used as standards for quality control tracking of binding antibody multiplex assay (BAMA) and biolayer interferometry (BLI) avidity assays performed over several days.

#### BAMA

CSP- and HepB-specific binding antibody responses in MAL068 or MAL071 participant serum or plasma were assessed as described previously (Tomaras et al., 2008; Yates et al., 2011; Yates et al., 2013). We evaluated antibody binding to full-length CSP, NANP6, NPNA3, C-term, and HepB. Briefly, vaccinee sera were diluted in BAMA assay diluent (1% milk-blotto, 5% normal goat serum, 0.05% Tween-20) and incubated with antigen-coupled microspheres. Samples were incubated with either anti-human IgG1 (BD Pharmingen, clone 12G8G11, anti-human IgG2 (Southern Biotech, clone HP6002), anti-human IgG3 (Invitrogen, clone HP6047), or anti-human IgG4 (BD Pharmingen, clone JDC-14), and detected on a Bioplex 200 (Bio-Rad). Controls for assays included a titrated purified human subclass specific standard curves or antigen-specific monoclonals and purified subclass-specific coupled beads. Negative controls in each assay included normal human reference serum (Sigma-Aldrich) and blank (no-antigen) beads. Each experiment was performed using Good Clinical Laboratory Practice–compliant conditions, including tracking of positive controls by Levey-Jennings charts. Mean fluorescence intensity (MFI) values were multiplied by dilution factor to adjust for different dilutions being used to get MFI values within the linear range of the assay. Positive responders were defined as samples with MFI >100, MFI*Dilution Factor >95th percentile of all baselines within study, and MFI*Dilution Factor > 3x baseline. Antibody avidity: Assessment of antibody avidity index (AI) was determined by BAMA with the following modifications: After formation of antigen/antibody immune complexes, a 15 min dissociation step (Na-Citrate, pH 4.0, Teknova; CIT) (Duong et al., 2012) at room temperature (20–23°C) was included prior to addition of secondary detection antibody. Retained binding magnitude was expressed as avidity index [AI; AI = MFI (CIT)/MFI (PBS)*100] and used as a measurement of antibody avidity in the statistical models. AI was calculated only in cases where binding response was positive according to pre-set criteria above. For multivariate analyses, AI was set to 0 for negative responses. Avidity index was reported for samples in the linear range where AI confirmed within 10% across assays and/or sample dilution factors. Samples that did not meet the pre-set criteria were reported as indeterminant for AI measurements.

#### BLI Avidity Assay

The BLI assay for monitoring the avidity of malaria vaccine-induced antibodies described previously (Dennison et al., 2018) was used to measure the RTS,S/AS01-induced serum antibody binding responses in MAL068 or MAL071 participants and the off rates of interaction with CSP-FL, NANP6, NPNA3, C-term, and N-interface. Antigens NANP6, PF16 and negative control peptide C1 were loaded onto streptavidin biosensors (threshold level set to not exceed Δλ = 1 nm) where as CSP-FL and negative control ovalbumin were coupled to the amine reactive (AR2G) biosensors (threshold level set to not exceed Δλ = 0.7 nm). The 1:50 diluted vaccinee sera binding to the parallel reference sensors immobilized with negative control antigens were subtracted to obtain antigen specific binding time courses. Binding responses (Δλ averaged at the last 5 s of association phase) and the off rates of vaccinee sera binding were determined. Antigen specific positivity limits (mean plus three times standard deviation of reference human serum binding response) and lower limits of quantitation (LLOQ; empirically determined antigen specific binding response above which off rate can be measured reliably for standard antibody) were applied in quality controlling of data. This involved ensuring that the percent coefficient of variation (%CV) in binding responses that are positive for a given antigen was <20 and the variation in off rates were ≤2 fold for sera with responses greater than LLOQ. For subclass assay, antigen specific antibodies were purified from vaccinees serum and were selectively captured onto IgG subclass specific antibody immobilized biosensors. Antigen binding was monitored by dipping the IgG subclass loaded sensors into wells containing CSP followed by dissociation in buffer. For univariate analyses, positive responders with binding responses below LLOQ were assigned an off rate of 1 × 10^−2^ s^−1^. For multivariate analyses, all vaccinees with binding responses below LLOQ were assigned an off rate of 1 × 10^−2^ s^−1^. Avidity Score, which encompasses both magnitude and avidity of response, is calculated as Score = response/off rate using the mean response and off rate values across triplicate measurements.

### Immune Response Biomarker Down-Selection

#### Low-Dimensional Assays

We use “low-dimensional” to refer to all immunoassays besides RNA-seq. For such assays, immune response biomarker down-selection consisted of three steps: 1) Initial data processing to collect all data for analysis; 2) Quantification of immunogenicity by running the model lmer[log10(magnitude) ∼ visit + arm + age + sex + (1 │ participant)] using R package lme4, pooled across arms and 3) Selection of measures with evidence of vaccine-induced response (FDR-adj *p* value < 0.1).

#### RNA-Seq

To use RNA-seq data [previously published in Du et al. (2020)] in the correlates analysis, the BTM framework (Chaussabel et al., 2008) was used with the BTMs published in Li et al. (2014). Assay-specific pre-processing consisting of standard normalization was performed. Gene-set enrichment analysis (GSEA) was performed for 345 BTMs, where each BTM consisted of the gene-set, to identify BTMs significantly associated with vaccination. The GSEA was performed using Bioconductor’s limma (camera). BTMs were collapsed into a single value for each participant from constituent genes using a weighted average, where the weights are log-transformed immunogenicity *p* values. All BTM expression values were calculated relative to Day 0 (baseline), for each participant. The following immunogenicity model was used: ∼Arm + Day + Sex + Age. *P* values and FDR-corrected P values were calculated with the limma package. For down-selection of the 345 BTMs, we selected BTMs with significant immunogenicity on day of challenge (FDR *p* value < 0.10).

#### Numbers of Clinical and Genomic Features Included in the Analysis

After following the procedures above, the following numbers of features were included in the analysis: MAL068 low-dimensional: 208 features filtered down to 120 for analysis; MAL068 RNAseq: 15131 genes mapped to 259 BTMs, filtered down to 155 for analysis; MAL071 low-dimensional: 587 features filtered down to 129 for analysis; MAL071 RNAseq: 15684 genes mapped to 259 BTMs, filtered down to 129 for analysis; MAL068 and MAL071 combined, low-dimensional: 140 features filtered down to 87 for analysis; MAL068 and MAL071 combined, RNAseq: 15084 genes mapped to 259 BTMs, filtered down to 133 for analysis.

### Predictive Modeling

#### Single-Variable Analysis

After biomarkers were down-selected, they were collected together for each study separately and each measure was standardized to have mean 0 and standard deviation one for accurate comparison of effects. Each biomarker was then used individually in a logistic model to evaluate association with protection, controlling for arm. The following model was used: infection ∼ biomarker + arm. From the model the odds-ratio associated with a unit change in the biomarker was collected along with a 95% confidence interval and *p*-value. *P*-values were adjusted across each study using the Benjamini-Hochberg procedure to control the false discovery rate and variables were considered significant if the adjusted *p* value was < 0.2.

Cross-validated area under the receiver operating characteristic curve (CV-AUC) values for each biomarker were calculated by splitting the target into a 5-fold cross-validation set, training the above logistic model on 80% of the data and predicting the held-out 20% for each fold. The resulting predictions were then used to calculate an AUC value. This was repeated 200 times with random partitions of the data to obtain a range of AUC values, and the cvAUC package was used to calculate the overall cross-validated AUC value.

Principal components analysis was done for each study using all down-selected variables. Missing data was imputed using the impute package in R prior to principal component analysis.

#### Principal Component Analysis

All biomarkers that passed immunogenicity down-selection were included in the principal component analysis. Imputation was done using impute.knn in R to fill any missing data, and each marker was centered and scaled. PCA was performed using prcomp in R.

#### LASSO

For LASSO (Friedman et al., 2010), cv.glmnet from the glmnet package was used. LASSO was run using s = “lambda.min”.

#### Double- and Triple-Variable Analyses

Analysis with multiple variables was done in the same manner as the single-variable analysis but examined additive models of all combinations of two and three variables, leading to a combinatorial explosion in the number of models considered. Both the logistic model and cross-validated AUC were evaluated for each model.

#### Cross-Study Analysis

To evaluate the cross-study consistency of measured biomarkers, models were trained on MAL068 (either only the RRR arm, or all data: RRR + ARR arms) and validated on the MAL071 RRR arm using markers measured in both studies. Because the studies share only one vaccine regimen, arm was not controlled for in the logistic model. Outcomes in MAL071 RRR were predicted using models fit on MAL068 (RRR or full), and an AUC was calculated for each model. All 1, 2, and 3-variable models were evaluated in this manner.

## Results

### Predicting Individual-Level Challenge Outcomes Using Day-of-Challenge Immune Response Data

#### Overall Analysis Strategy

**FIGURE 2:**
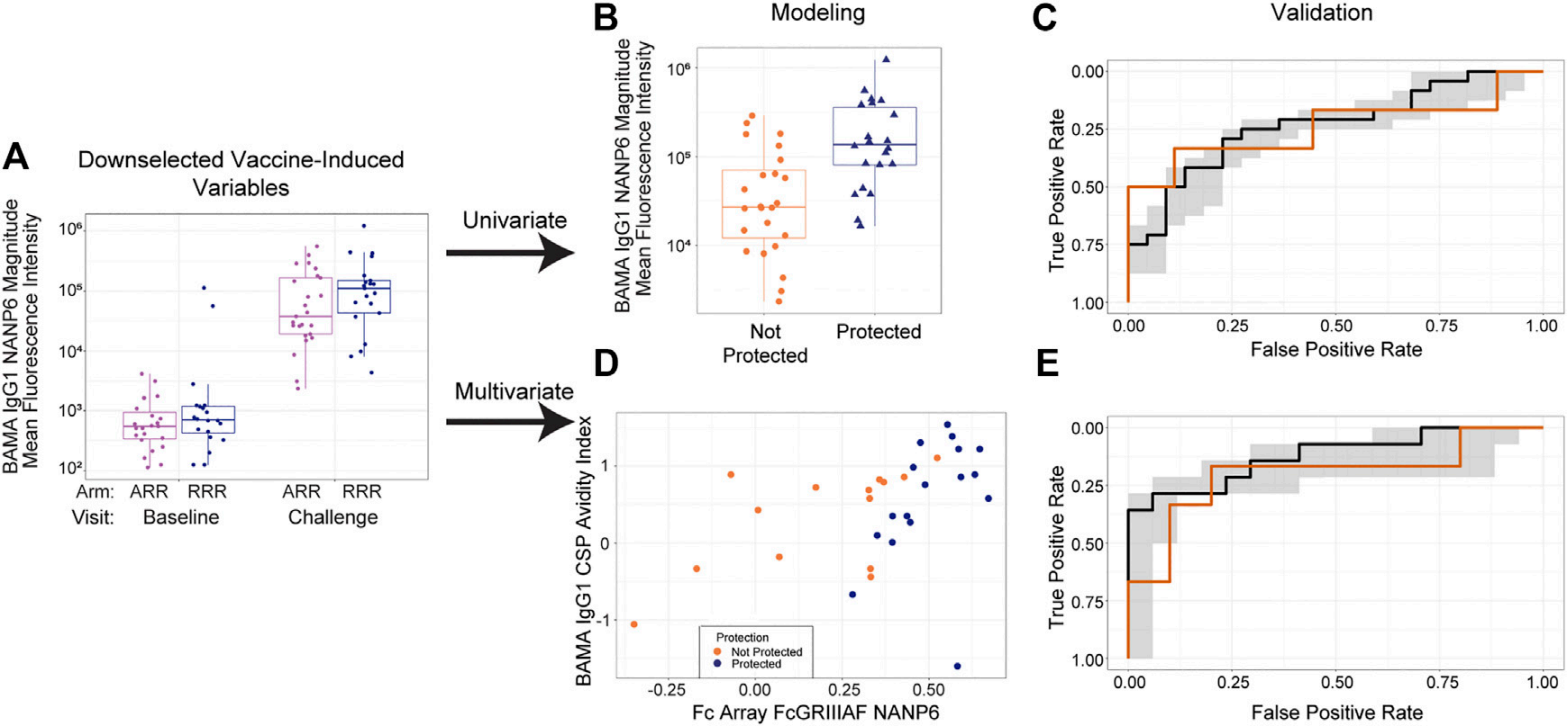
Overall analysis strategy, consisting of **(A)** down selection, **(B)** univariate modeling followed by **(C)** validation and **(D)** multivariate modeling followed by **(E)** validation. **(A)** Boxplot showing IgG1 anti-NANP6 binding antibody responses at baseline and at challenge in MAL068. Purple dots = ARR arm; blue dots = RRR arm **(B)** Boxplot showing day of challenge IgG1 anti-NANP6 binding antibody responses separately by post-challenge outcome status (not protected vs protected) in MAL068. **(C)** Univariate model trained on all MAL068 data predicting challenge outcomes in the MAL071 RRR arm as a function of day of challenge IgG1 anti-NANP6 binding antibody responses **(D)** Scatter plot of day-of-challenge BAMA IgG1 CSP Avidity Index and Fc Array FcGRIIIAF NANP6 binding in MAL068, with points colored by protection status (orange = not protected, blue = protected). **(E)** Best-performing two-variable additive model trained on all MAL068 data predicting challenge outcomes in the MAL071 RRR arm as a function of day of challenge Fc Array FcGRIIIAF NANP6 binding and BAMA IgG1 CSP Avidity Index. In panels **(C)** and **(E)**, the black line is the median ROC curve from cross-validation on all MAL068 data, the gray shaded area is the 95% empirical interval for the ROC curve from cross-validation on all MAL068 data, and the orange line is the ROC curve for predicting MAL071 RRR arm data. BAMA = binding antibody multiplex assay.

Figure 2 shows the overall analysis strategy for how we assessed the ability of immune response variables to predict challenge outcomes. Given the very large number of candidate immune response variables that could be assessed within each data set for contribution to prediction of RTS,S/AS01-mediated protection, immune response down-selection based on favorable statistical properties (e.g., high reproducibility, large dynamic range of vaccine-induced responses) was crucial for first narrowing down these responses and improving statistical power. As the trial outcomes are known, we could not conduct a traditional down-selection. Instead, using unblinded assay data from each data set, we down-selected immune response variables based on immunogenicity (i.e. vaccine-induced response on day of challenge; statistical details are given in Methods). Figure 2A shows an example immunogenicity plot for IgG1 anti-NANP6 binding antibodies in MAL068, where a clear vaccine-induced response can be seen in each study arm. A full list of down-selected immune response variables for the MAL068 data set and for the MAL071 data set can be found in Supplementary Table S1.

We explored univariate (Figures 2B,C), bivariate (Figures 2D,E), and trivariate (not shown) modeling approaches. For the univariate approach (Figure 2B), we ran a model predicting the challenge outcome as a function of each variable that passed immunogenicity down-selection, separately for MAL068 and for MAL071, controlling for study arm. As a complementary approach (Figure 2D), we explored all possible 2-variable and 3-variable models to see if prediction would be improved by including multiple immune response biomarkers. To look at the cross-study replicability of the findings of each approach, we validated the trained models by evaluating each model on a test data set including data from the other study.

### Top Variables in Predicting MAL068 Challenge Outcomes Include NANP6-Specific Binding Antibodies, Fc Array Variables Related to FcgR Specificity of NANP6-Specific Antibodies, and NANP6-Targeted ADCP

**FIGURE 3:**
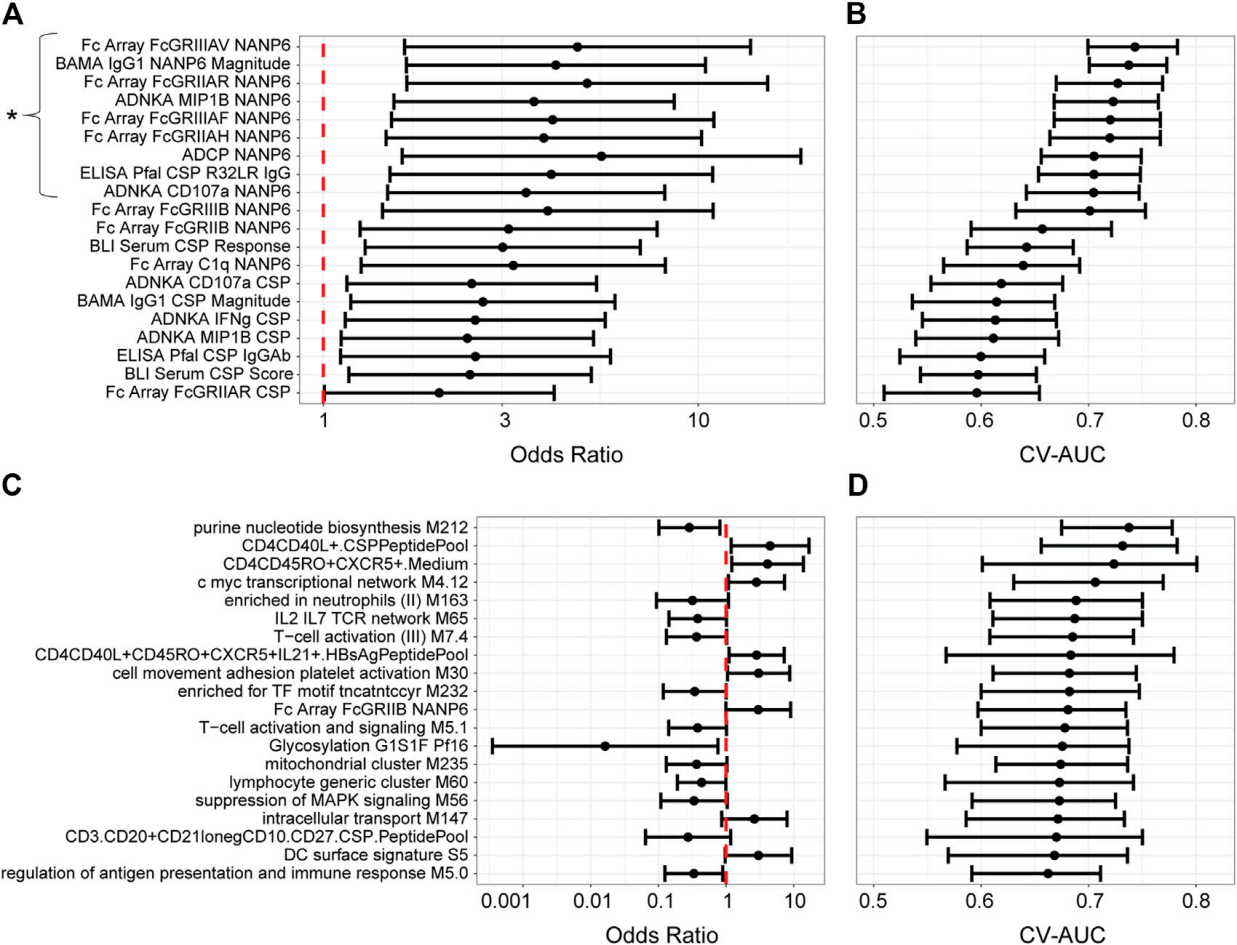
Univariate models with MAL068 (ARR + RRR) and with MAL071 (RRR + RRr) data. **(A, C)** Odds ratios for protection against patent parasitemia per unit increase in the designated immune response variable on the day of challenge, for the 20 most predictive immune response variables in each data set (A, MAL068; C, MAL071). Whiskers extend through the 95% CI. The vertical dashed red line indicates an odds ratio of 1, i.e., that protection is equally likely whether the designated immune response is higher or lower on challenge day. The asterisk in panel A designates the nine variables that were significantly associated with protection, having *p*-values < 0.2 after adjustment for false discovery rate. No variables were significantly associated with protection in panel **(C)**. **(B, D)** Forest plot of cross-validated AUC scores for the different univariate models for each data set (B, MAL068; D, MAL071). Dots show the cross-validated AUC calculated using the cvAUC package in R; whiskers extend through 95% empirical intervals of the 200 individual 5-fold cross-validation runs.

For the univariate models (Figure 3), the results are expressed as odds ratios (ORs) for post-challenge protection against patent parasitemia per unit-increase in a given immune response variable on challenge day (i.e. correlation of that immune response variable with protection) and CV-AUC (i.e. predictive capacity of that immune response variable, also a more robust measure since it uses a training set and predicts on the held-out data). A CV-AUC of 1.00 indicates perfect prediction, while a CV-AUC of 0.50 indicates prediction equivalent to random chance.

The results differed substantially between MAL068 and MAL071. For MAL068 (RRR and ARR groups pooled), immune responses related to antibody Fcγ receptor specificity and to the NANP6 repeat region were highly represented in the top 20 most predictive variables (Supplementary Table S2). Nine immune response variables (eight of which were related to NANP6) were significantly associated with protection (FDR-adjusted *p*-value ≤ 0.2 and *p*-value ≤ 0.05) and showed fair performance in predicting challenge outcome across the two groups pooled (controlling for group/arm) (CV-AUC ≥ 0.7). In contrast, no blood transcriptional modules (BTMs) ranked among the top 20 most predictive variables. This finding is consistent with Kazmin et al. observation that the robust transcriptional responses observed after the three vaccine doses in MAL068 had almost completely waned by day of challenge in both arms (Kazmin et al., 2017), suggesting loss of signal for detecting transcriptional correlates in peripheral blood on day of challenge.

In MAL071 (RRR and RRr groups pooled), the majority (*n* = 14) of the top 20 most predictive variables in MAL071 were BTMs; however, no immune response variable was significantly associated with protection, perhaps underscoring the difficulty in identifying transcriptional correlates on day of challenge or reflecting that the two arms may have different mechanisms of protection. The only variable that ranked in the top 20 most predictive variables of both trials was Fc Array FcGRIIB NANP6 [CV-AUCs: MAL068 = 0.660 (0.590–0.720), MAL071 = 0.680 (0.600–0.730)]. Moreover, in MAL068, the top 20 variables all associated with protection (OR > 1), whereas in MAL071, less than half (*n* = 8) of the top 20 variables associated with protection and the remaining (*n* = 12) associated with non-protection, albeit none significantly after false discovery rate (FDR) adjustment.

To examine groupings between different immune response variables, particularly across assay types, and thus potentially gain insight relationships between the different components of the immune response, we computed pairwise correlations across all variables in the MAL068 analysis (RRR and ARR arms; Supplementary Figure S1; top-20 ranked, Supplementary Figure S2). The results showed that many of the top-ranked variables were highly correlated (e.g., R > 0.9 for nearly all the NANP6 Fc array variables). The strong correlations of many of the top-performing variables raised the possibility that they may be reflecting a coordinated component of the immune response, e.g. in the same way that significant correlations across diverse Fc effector assay responses have supported a coordinated antibody response in recruiting innate immune cells in elite controllers of HIV-infection (Ackerman et al., 2016). The strong correlations also likely reflect the particular NANP6 antibody subclass (i.e., IgG1) that is needed to engage with each of the Fc receptors for a functional anti-parasitic response — and also ties in with BAMA IgG1 NANP6 Magnitude scoring highly as a predictor in MAL068. For the MAL071 analysis (correlations for all variables, Supplementary Figure S3; correlations for top-20 ranked variables, Supplementary Figure S4), the top 20 variables seemed to be somewhat less highly correlated with each other, and high inverse correlations were also observed (R < −0.8).

### A Fully Unsupervized Approach did not Show Discrimination Between the Protected and Non-Protected Groups of Either Trial

We next performed a principal component analysis (PCA) on the data from each study to assess whether a fully unsupervized approach would show discrimination between the protected and non-protected groups of each trial and, if so, which variables contribute to such discrimination. The top 20 variables of each PC for MAL068 (RRR and ARR arms) and MAL071 (RRR and RRr arms) are listed in Supplementary Table S3.

**FIGURE 4:**
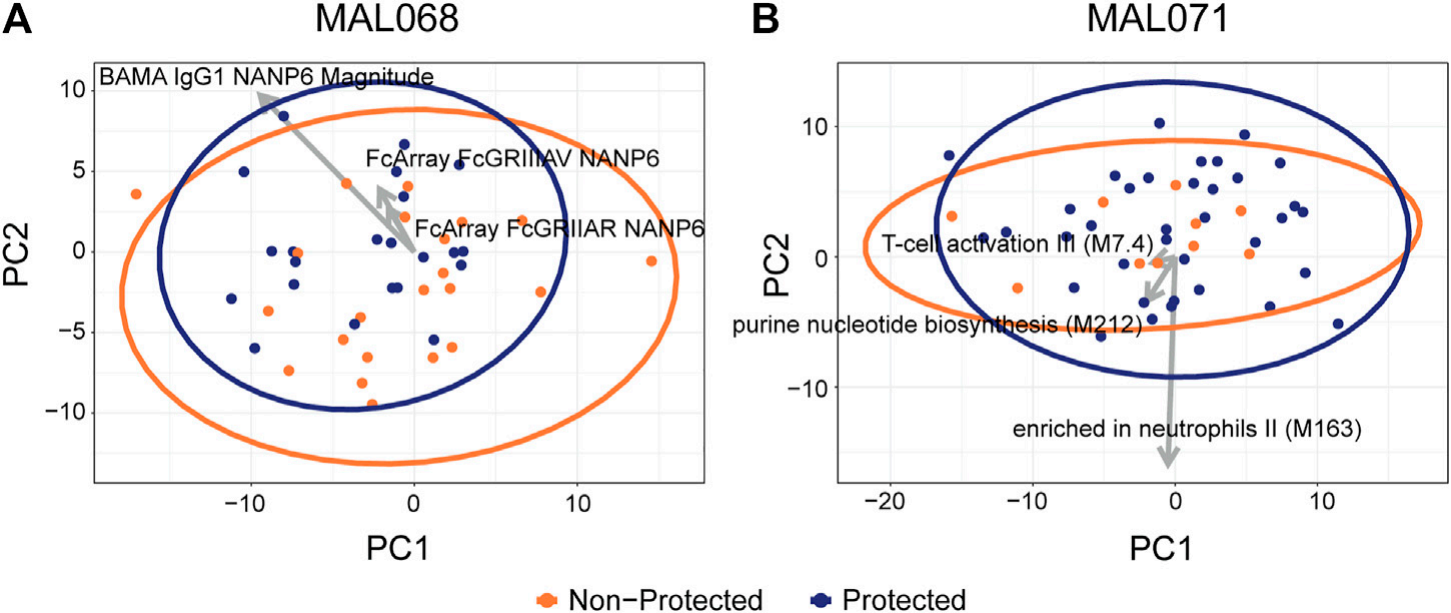
Scatterplots of participant factor scores on the first (PC1) and second (PC2) principal components of a principal components analysis on **(A)** MAL068 data (ARR and RRR arms); **(B)** MAL071 data (RRR and RRr arms). Arrows show the factor loadings for the top three variables by CV-AUC for each dataset. A total of 220 features were used in the PCA analysis, 133 of which are RNA-seq BTMs.

The PCA of MAL068 showed no discrimination between the protected and non-protected groups on principal component (PC) one or PC2 (Figure 4A). PC1 accounted for 25% of the total variation. Its top variables were all BTMs, five of which related to monocytes. PC2 accounted for 13% of the total variation. Its top variables included CSP-specific, C-term-specific, and some NANP6-specific antibody features spread across a range of functional assays.

Like the MAL068 results, the PCA of MAL071 did not show any discrimination between the protected and non-protected groups. PC1 accounted for 26% of the total variation; most of its top variables were CSP-specific or C-term-specific Fc array variables (Supplementary Table S3). PC2 accounted for 10% of the total variation and its top variables were nearly all BTMs.

### NANP6-Specific Antibody Titer, Fc Array Variables Related to FcγR Specificity of NANP6-Specific Antibodies, and NANP6-Specific ADCP Contribute to Cross-Study Prediction and are Associated With Protection

Before examining the cross-study replicability of our findings, we considered the substantial difference between the MAL068 and MAL071 results thus far. A possible reason for this difference is that the two trials compared different regimens, the protective mechanisms of which may have differed [as suggested by the findings of (Chaudhury et al., 2017; Kazmin et al., 2017; Pallikkuth et al., 2020; Suscovich et al., 2020)]. These findings, coupled with the higher efficacy of the RRr arm in preventing patent parasitemia (VE of the RRr arm = 87% in MAL071 vs VE of the RRR arm = 52% in MAL068, 63% in MAL071), prompted us to restrict our cross-study validation to predicting RRR outcomes in MAL071.

**FIGURE 5:**
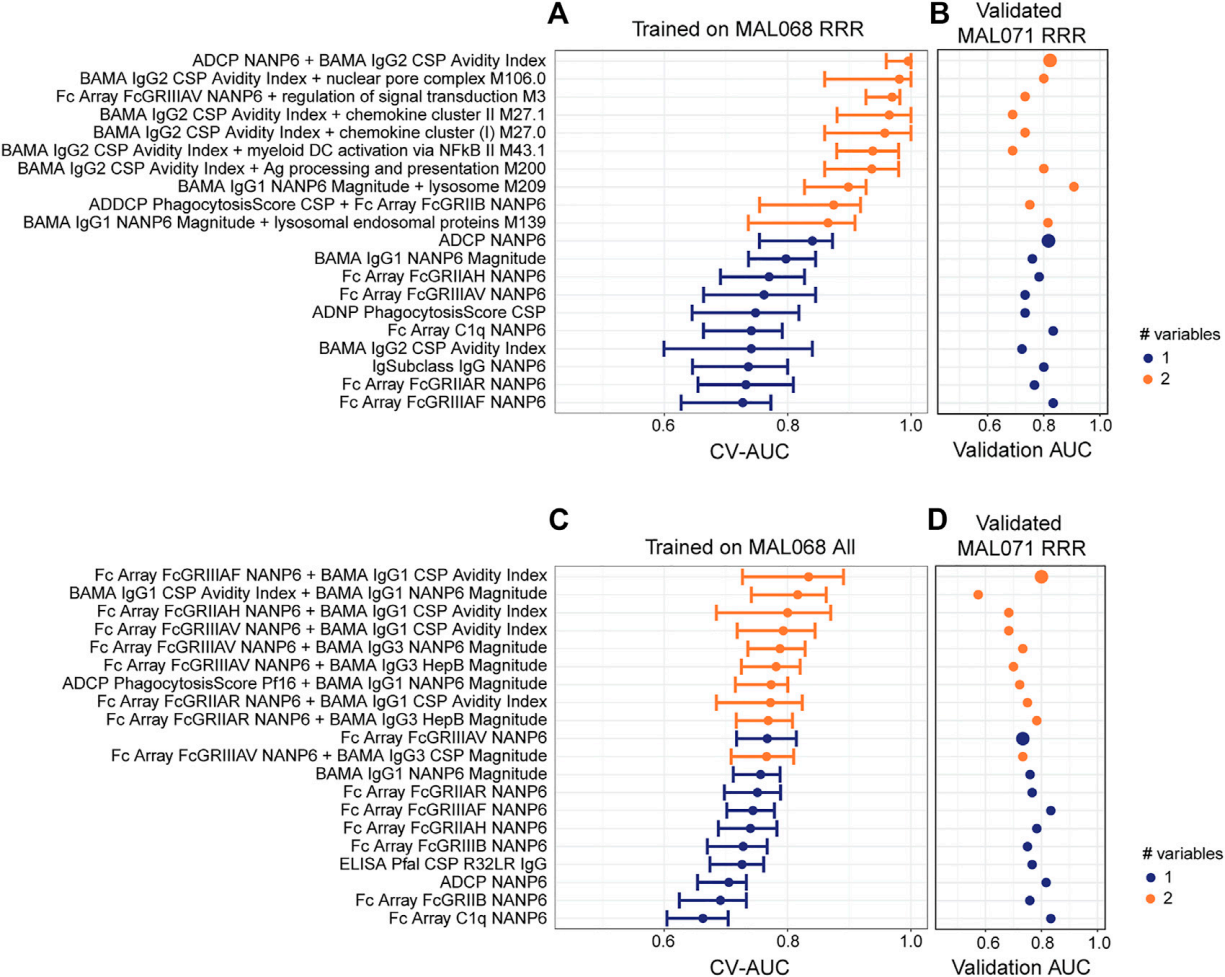
Cross-study validation of univariate and 2-variate models. **(A, C)** Forest plots of cross-validated AUC scores for the top-performing univariate and 2-variate models trained on **(A)** the MAL068 RRR data set or **(C)** the entire MAL068 data set. All data were day-of-challenge. Dots show the cross-validated AUC calculated using the cvAUC package in R; whiskers extend through 95% empirical intervals of the 200 individual 5-fold cross-validation runs. **(B, D)** Validation AUCs of the same models as evaluated in predicting post-challenge outcomes in the RRR arm of MAL071. Purple, single-variable model; orange, two-variable model. The slightly enlarged bubbles in **(B, D)** identify the top-performing models as assessed by CV-AUC **(A, C)**.

For the cross-study validation, all possible univariate and bivariate models were trained on MAL068 (either the RRR data set, or the entire data set) and validated on the MAL071 RRR data set. Figure 5 shows the CV-AUC values for the top-10 performing univariate and top-10 performing bivariate models for the MAL068 RRR data set (panel A) and for the entire MAL068 data set (panel C). It also shows the validation AUC for each model when validated on the MAL071 RRR data set (panels B and D). The CV-AUC and validation AUC values are provided in Supplementary Table S4, along with the direction of association of each variable (protection vs non-protection).

Regardless of whether the models were trained on MAL068 RRR or on the full MAL068 dataset, the bivariate models in the training set almost always had higher CV-AUC values than the univariate models, whereas little or no improvement in validation AUCs was observed in the validation dataset for the bivariate models compared to the univariate models. For example, the top-performing (CV-AUC = 0.996) bivariate model trained on MAL068 RRR (ADCP NANP6 + BAMA IgG2 CSP Avidity Index) includes variables that were each identified in the top 10-performing univariate models (first-best and seventh-best, respectively). As assessed by validation AUC, the ADCP NANP6 + BAMA IgG2 CSP Avidity Index model (0.822) showed no appreciable improvement over the univariate ADCP NANP6 model (0.817) (Figure 5, Supplementary Table S4). This trend held true across the other bivariate models, none of which showed any appreciable improvement over the corresponding univariate models in cross-study prediction, with the exception of BAMA IgG1 NANP6 Magnitude + lysosome M209, whose validation AUC (0.907) exceeded those of all the univariate models (highest validation AUC = 0.833; Figure 5B, Supplementary Table S4). Similar results were obtained for models trained on the entire MAL068 data set, where no improvement in cross-study prediction was achieved with the top-performing bivariate model (Fc Array FcGRIIIAF NANP6 + BAMA IgG1 CSP Avidity Index, validation AUC = 0.800) compared to the univariate Fc Array FcGRIIIAF NANP6 model (validation AUC: = 0.833). We conclude that, using these datasets, cross-study outcomes can be reasonably well predicted within the RRR arm, but that there is little or no benefit in including more than one immune response variable in the predictive model. It is possible that future studies with more participants (and a relatively even case:control split) might allow for more complex models.

Looking across the variables that tended to be identified in top-performing models, we find that many variables were related to NANP6 and were associated with protection (Supplementary Table S4). For models trained on MAL068 RRR, Fc array variables related to the specificity of FcγR binding of NANP6-specific antibodies were consistently associated with protection, along with BAMA IgG1 NANP6 Magnitude, ADCP NANP6, and BAMA IgG2 CSP Avidity Index. The few variables associated with non-protection were mostly all BTMs. For models trained on the full MAL068 dataset, Fc array variables related to the specificity of FcγR binding of NANP6-specific antibodies were also consistently associated with protection, again along with BAMA IgG1 NANP6 Magnitude and ADCP NANP6. The variable most often associated with non-protection was BAMA IgG1 CSP Avidity Index, which associated with non-protection in five of the bivariate models. These results are consistent with those shown in Figure 3 and again suggest that NANP-specific ADCP, NANP-specific IgG1 binding antibodies, and Fc array variables related to the specificity of FcγR binding of NANP6-specific antibodies are associated with protection. The cross-study prediction also highlights BAMA IgG2 CSP Avidity Index as consistently associated with protection across models, and BAMA IgG1 CSP Avidity Index as consistently associated with non-protection across models.

Performing the same analysis but predicting MAL071 outcomes in the RRr arm showed poorer predictive performance (i.e., lower validation AUCs) of the top-performing models (Supplementary Figure S5). The reduced predictive performance could be due to differences in mechanism of protection, or the higher VE in the RRr arm, leading to a relative imbalance of protected over nonprotected participants. Consistent with the MAL071 RRR cross-study prediction results, the MAL071 RRr cross-study prediction also showed little to no benefit of bivariate vs. univariate models.

When we trained models on MAL071 (RRR or both arms combined) and validated on MAL068 (RRR or both arms combined), the results were essentially the same, except that MAL071 showed a large number of RNAseq variables that ranked highly in terms of CV-AUC on MAL071, and the RNAseq variables were not consistent when applied to MAL068 (data not shown).

### Complementary Approaches Yield Consistent Results to Those Obtained by our Approach

We next explored some complementary approaches to see whether they would yield consistent results to those obtained by the workflow outlined in Figure 2. First, we took a leave-one-out cross-validation strategy on the MAL068 RRR cohort for comparison with our framework. The results were very similar, with a correlation between the resulting AUC values of 0.96, supporting our approach. Next, we used LASSO to build a model using the MAL068 RRR cohort, and then used this model to predict MAL071 RRR outcomes. The model chosen by LASSO included two variables: BAMA IgG1 NANP6 Magnitude and Fc Array FcGRIIIAV NANP6. These are the variables ranked two and four by our method (see Supplementary Table S4A). The validation AUC on MAL071 RRR for the LASSO model was 0.678. For comparison, for models trained on MAL068 RRR in our approach, the bivariate model with the top validation AUC (predict on MAL071 RRR) was BAMA IgG1 NANP6 Magnitude + lysosome M209 (validation AUC 0.907) and the univariate model with the top validation AUC (predict on MAL071 RRR) was Fc Array FcGRIIIAF NANP6 (validation AUC 0.833) (Supplementary Table S4A), thus validating our approach.

### No Improvement in Cross-Study Prediction in Bi- or Tri-Variate Models Compared to Univariate Models, for the Dataset Used in This Analysis

To further explore whether including additional immune response variables could improve cross-study model prediction, we plotted CV-AUC for the top 200 univariate, bivariate, and trivariate models trained on MAL068 (all data or RRR) vs. the validation AUC on MAL071 data (all, RRR, or RRr) (Supplementary Figure S6). While the trained CV-AUCs generally increased from uni- to bi- and again from bi- to trivariate models, no improvement was seen in validation AUC for bi- or trivariate models compared to the univariate models, across all training sets and across all validation sets. These data reinforce the conclusion that including more variables in the predictive models does not improve cross-study prediction, at least for the data pre-processing method used in our approach. The figure also shows overfitting of the trivariate models, with all trivariate models in each training/validation set combination tightly clustered in a narrow band just next to the perfect CV-AUC value of 1.

## Discussion

Building upon the wealth of previous RTS,S/AS01 immune correlates analyses, here we took a predictive modeling approach that included immune response data across many different assays and assessed the contribution of each immune biomarker to prediction of individual-level outcomes of RTS,S/AS01 vaccinees in two different controlled human malaria infection studies. We identified univariate and bivariate models with good predictive accuracy across studies [validation area under the receiver operating characteristic curve (AUC) > 0.8], and our first major conclusion is that there was little to no improvement in the bivariate models compared to the univariate models, when combining all the day-of-challenge biomarker data into a comprehensive dataset. We hypothesize that the relatively small sample sizes, which in turn limited the size of the training/testing/validation sets, hindered the identification of well-performing complex models. Applying the same approach to future studies with larger sample size may enable such models to be identified. Another potential explanation for the lack of significant improvement in bivariate vs univariate models (when combining all day-of-challenge biomarker data into a comprehensive dataset) is the high correlation between variables, as highly correlated variables will generally not improve in logistic regression over single variable models.

We identified central NANP repeat region (NANP6)-targeted antibody dependent cellular phagocytosis (ADCP), binding of NANP6-targeting antibodies to the FcGRIIIa receptor, and anti-NANP6 IgG1 antibody titer as consistently predictive immune response measurements, with higher levels of each biomarker on day of challenge associated with individual-level protection after controlled malaria challenge. Our second major conclusion is thus that our data support a “quality as well as quantity” hypothesis for how RTS,S/AS01-induced antibodies may protect, in line with the previous findings of Suscovich et al. (2020) (discussed further below). Regarding full-length CSP-specific antibodies, we found contrasting associations of avidity with protection, depending on antibody subclass (IgG1 with non-protection; IgG2 with protection).

We next relate our results to the published correlates analyses of MAL068 and MAL071 that were introduced earlier. In the systems serology analysis by Suscovich et al. (2020), LASSO was used to downselect from a broad array of qualitative and quantitative antibody variables measured on the day of challenge in MAL068 vaccinees, build multivariate models, and assess how well each model could discriminate protected from non-protected vaccinees. Similar to our findings, Suscovich et al. reported that parsimonious models were able to accurately discriminate protected from non-protected vaccinees across studies, with a bivariate model (trained on MAL068 RRR) containing NANP6-specific ADCP and FCGRIIIA binding of NANP6-specific antibodies well-predicting protection in both the RRR arm of MAL071 and in the RRR arm of MAL027, another controlled human malaria infection study (Kester et al., 2009). While none of the top-performing bivariate models in our analogous cross-study validation (built on MAL068 RRR and validated on MAL071 RRR) contained the exact same combination of variables as the model in Suscovich et al. (ADCP NANP6 and FCGRIIIA NANP6), FcGRIIIAV NANP6, FcGRIIIAF NANP6, and ADCP NANP6 all ranked highly in our top-performing univariate and bivariate models. Therefore, our results are broadly consistent with the conclusion of Suscovich et al. that NANP6-specific antibody engagement of multiple effector cell types may be important for protection. However, our findings differed from Suscovich et al. in that we identified NANP6-specific antibody engagement of FcGRIIA, FcGRIIB, and FcGRIIIB as associated with protection in our top-performing models in the cross-study validation, whereas the FCGRII-related variables (NANP6 FcGRIIA, NANP6 FcGRIIB) were not among the five-feature LASSO-selected model, nor the two-feature model, reported by Suscovich et al. to discriminate protected from nonprotected participants, nor were NANP6 FcGRIIA, NANP6 FcGRIIB, or NANP6 FcGRIIIB found to be significantly different in univariate profiling of protected vs nonprotected participants by Suscovich et al. FcGRIIA is expressed by neutrophils and mediates antibody-dependent cellular cytotoxicity (ADCC) (Derer et al., 2014; Treffers et al., 2018) and is also expressed by monocytes, dendritic cells and macrophages and mediates ADCP (Anania et al., 2019); FcGRIIB is an inhibitory FcγR expressed by B cells (Amigorena et al., 1989), dendritic cells, macrophages, activated neutrophils, mast calls and basophils (Nimmerjahn and Ravetch, 2008) and FcGRIIIB is expressed by neutrophils and mediates endocytosis of immune complexes (Chen et al., 2012). The shared expression of FcGRIIA and FcGRIIIB by neutrophils suggests a potential role in NANP6-targeted ADNP in protection, which is consistent with the network linkage of NANP6 ADCP with ADNP in Suscovich et al.

Moreover, consistent with the findings of Seaton et al. (under revision)^1^, NANP6-specific IgG1 scored highly in the top-performing models. IgG1 antibodies are known to bind to FcGRs, including FcGRII and FcGRIII and subsequently mediate downstream effector functions such as ADCC and ADCP. Taken together, these data indicate a role for NANP6-specific antibody activation of multiple FcγR-expressing immune cell effector functions. While we did not identify ADCC NANP6 in any of our top-performing models, the identification of NANP6-specific FCGRIII-related Fc array variables in our top-performing models suggests that effector functions beyond phagocytosis may be important.

Dennison et al. recently analyzed antibody avidity data from MAL071 and reported that, while no association with protection was seen with the avidity of antibody responses to CSP, NANP6, C-term, or NPNA3 in the RRR arm, higher-avidity antibodies to CSP and C-term tended to associate with protection in the RRr arm, suggesting a potential difference in the mechanisms of protection in the two arms. Our findings are consistent with those of Dennison et al. in that BLI variables related to avidity of anti-CSP or anti-C-term antibodies did not appear in top-performing (as assessed by cross-validated AUC) models trained on MAL068 RRR.

We next discuss our findings in the context of the previous transcriptional correlates studies (Kazmin et al., 2017; Du et al., 2020). A major difference between our study and the previous studies is that ours is the first to have included day-of-challenge PBMC transcriptional data in a correlates analysis. Kazmin et al. identified relatively early (within one week) transcriptional signatures of protection in the ARR and RRR arms of MAL068 and reported day-of-challenge functional enrichment (compared to baseline) for many BTMs in both study arms, but did not perform the correlates analysis on day-of-challenge levels. Likewise, Du et al. focused on the 1 day post-third dose time point, which falls within the innate response window (Du et al., 2020), and complements our focus on the day of challenge, at which point the innate adjuvant-driven response has waned (Kazmin et al., 2017). While we identified only a few BTMs in our top-performing models, two of the top three two-variable models (in terms of validation AUC on MAL071 RRR) each included a lysosome-related BTM (“lysosome M209” in the top-performing model and “lysosomal endosomal proteins M139” in the third-best performing two-variable model). Interestingly, day-of-challenge PBMC expression of each of these BTMs was associated with non-protection. The M209 BTM was recently proposed to represent the peroxisome (Courtland et al., 2021) based on high-resolution spatial proteomic data (Geladaki et al., 2019). There is now abundant evidence that peroxisomes regulate and influence both immune function and inflammation (Di Cara et al., 2019), and while peroxisomes have been shown to play an important role in antiviral signaling (Dixit et al., 2010), to our knowledge, an association between peroxisomes and malaria susceptibility has not previously been described. Further studies would be needed to confirm such an association.

Du et al. found that the discriminatory ability of the MX2/GPR183 signature varied over time, with little to no difference between log_2_ fold-change (vs baseline) MX2/GPR183 ratio in protected vs. nonprotected vaccinees on day of challenge. It is thus not surprising that we did not identify any BTMs related to MX2 (a prototypical interferon-induced gene) or GPR183 (an oxysterol receptor) among our top-predicting variables in our day-of-challenge analyses. However, similar to Du et al., we did find that our top-predicting variables were not correlated with the known correlate anti-CSP titer (Dobaño et al., 2019), suggesting that our study also identified a separate aspect of the RTS,S/AS01-induced immune response implicated in protection.

Pallikkuth et al. used machine learning to identify combinations of immune response biomarkers, focusing on B-cell and peripheral T follicular helper (pTfh) cell responses, that were predictive of individual-level protection in the MAL071 trial (Pallikkuth et al., 2020). While we only analyzed day-of-challenge biomarkers, Pallikkuth et al. also analyzed immune responses throughout the 3-dose vaccination schedule up to the day of challenge: many of their most predictive variables were from relatively early time points, e.g., Day 6 post-dose one and Day 28 post-dose 2 (these time points were designed to obtain samples at critical periods of follicular helper T cell development). In their best-performing model, which contained 18 parameters, 85% of all individual-level outcomes were predicted correctly. Their analysis thus complements ours and reinforces the conclusion that molecular signatures of protection can vary greatly depending on the specific time point(s) post vaccination that are examined.

Our focus on day-of-challenge immune response measurements meant that our models did not incorporate all previously identified correlates of protection, e.g., the many transcriptional modules whose early post-vaccination (pre-challenge) expression was found to associate with protection (or non-protection) reported by Kazmin et al. (2017), or the early CSP-specific pTfh responses reported by Pallikkuth et al. to associate with protection (Pallikkuth et al., 2020). We did not investigate here the extent to which these earlier responses linked to protection (or non-protection) correlate with our top-performing variables, and thus it is possible that some individuals may have mounted an earlier protective response whose transcriptional “stamp” did not persist until day-of-challenge. We conjectured that post-challenge outcomes of such individuals would be harder to predict, since they would be determined by immune responses not included in the analysis set used here. In a leave-one-out prediction analysis, we did find evidence that cross-study post-challenge outcomes are easier to predict (using a day-of-challenge dataset) for some participants than others (Supplementary Figure S7). In particular, in the validation set, individuals who became infected after challenge tended to be correctly predicted by higher proportions of models compared to individuals who did not become infected after challenge. The presence of a cluster of four individuals whose MAL071 RRR challenge outcomes were particularly poorly-predicted (< 25% of all models) by models built on MAL068 RRR is consistent with the hypothesis that immune response kinetics and/or qualities may have varied across participants, and that this subset may have mounted quicker protective responses that were not reflected in the day-of-challenge dataset used here.

Additional limitations of our work include that our study is in the context of a highly controlled challenge environment, and there are important differences between the CHMI studies [malaria-naïve adults, CHMI done using a three-dose schedule with a one-month interval (with the exception of the RRr arm)] and the current target population for which RTS,S/AS01 is licensed (young children in malaria-endemic areas, RTS,S/AS01 is recommended to be used as a four-dose regimen, including a booster dose). Thus, it remains to be seen whether the biomarkers highlighted by our study are similarly relevant biomarkers of protection in malaria-endemic field settings. However, in the light of our finding that anti-NANP6 IgG1 titer was consistently associated with protection, Dobaño et al. (2019) reported that the magnitude of anti-NANP6 IgG antibodies was significantly associated with vaccine efficacy against clinical malaria, and Ubillos et al. (2018) reported that the RTS,S/AS01-induced increase (baseline to Month 3) in anti-NANP6 IgG1 antibodies was associated with protection from clinical malaria, in subsets of participants drawn from the phase 3 efficacy study of RTS,S/AS01 in infants and young children in malaria-endemic regions in Africa (RTS, S Clinical Trials Partnership, 2011; RTS, S Clinical Trials Partnership, 2012; RTS, S Clinical Trials Partnership, 2014; RTS, S Clinical Trials Partnership, 2015). These findings provide some evidence that our findings may apply in diverse populations that differ in important respects, e.g., baseline malaria immune status, although correlates were not assessed in post-booster samples in either Dobaño et al. or Ubillos et al.

Second, the high efficacy of the RRr regimen in MAL071 hindered the development of models that could discriminate protected from non-protected individuals; thus, we had to restrict our cross-study analysis to the RRR arm of MAL071. Thirdly, by focusing on day of challenge, we likely missed the opportunity to pick up earlier immune response biomarkers–especially transcriptional modules–that may have waned by day of challenge. Additional limitations include the necessity of restricting our analysis to variables that were common across the two trials, the fact that we did not assess predictive ability of individual genes in addition to the BTM analysis, and the lack of inclusion of alternative gene sets [such as Gene Ontology (GO) gene sets related to e.g., immunological signatures (Godec et al., 2016) or to canonical pathways/processes (Liberzon et al., 2015)] in the gene set enrichment analysis. Despite these limitations, our study further highlights Fc effector-related attributes of NANP6- and subclass-specific antibodies, over all other immune features investigated, that merit further confirmation in field trials. Our study is thus valuable in potentially helping prioritize resource-intensive laboratory measurements for assessment in future studies.

## Data Availability

Publicly available datasets were analyzed in this study. This data can be found here: The MAL068 and MAL071 RNA-seq data analyzed in this study were published in Du et al. (doi: 10.3389/fimmu.2020.00669) and are available at Gene Expression Omnibus (accession numbers GSE103401, GSE102288, GSE107672). The MAL068 ICS, ELISpot, ELISA, and seroneutralization data published with Ockenhouse et al. can be accessed at www.gsk-clinicalstudyregister.com. Anonymized patient-level data underlying the MAL068 study will be made available to independent researchers, subject to review by an independent panel, at www.clinicalstudydatarequest.com. The MAL068 systems serology data were published with Suscovich et al. (doi: 10.1126/scitranslmed.abb4757). The MAL068 and MAL071 BLI and BAMA data are included in a article by Seaton et al. (under revision)^1^. MAL071 BLI data reported in Dennison et al. are available upon request at GSK. MAL071 ICS and ELISA data were described in Regules et al. (doi: 10.1093/infdis/jiw237 and are available upon request at GSK. Other MAL071 ELISA data and MAL071 Luminex data were published by Chaudhury et al. (doi: 10.1038/s41598-017-08526-5 and are available from the corresponding author upon request (Elke S. Bergmann-Leitner, elke.s.bergmann-leitner.civ@mail.mil). The MAL071 ICS, pTfh, and B cell phenotyping data were published with Pallikkuth et al. (doi: 10.7554/eLife.51889). Code: The primary analysis code is posted publicly at https://github.com/william-c-young/mal068_071_paper.

## Footnotes

1 Seaton, K. E., Spreng, R. L., Abraha, M., Reichartz, M., Rojas, M., Feely, F., et al. (2021). Subclass and Avidity of Circumsporozoite Protein Specific Antibodies Associate with Protection Status Against Malaria Infection. under revision

## Supplementary Material

SUPPLEMENTARY FIGURE 1 Pairwise correlations of day of challenge measurements among the 275 down selected immune response variables for the MAL068 full analysis (RRR and ARR) (i.e. the variables listed in Table S1). Cell color indicates the correlation between variables (scale bar at upper right of plot). The two columns on the left indicate the odds ratio for post-challenge protection against patent parasitemia per unit-increase in a given immune response variable on challenge day (“Odds Ratio”) and assay type (“Assay”). ADCC, antibody-dependent cellular cytotoxicity; ADCD, antibody-dependent complement deposition; ADCP, antibody-dependent cellular phagocytosis; ADDCP, antibody dependent dendritic cell phagocytosis; ADNKA, antibody-dependent natural killer cell activation; ADNP, antibody-dependent neutrophil phagocytosis; BAMA, binding antibody multiplex assay; BLI, biolayer interferometry; ELISA, enzyme-linked immunosorbent assay; ELISpot, enzyme-linked immunospot; ICS, intracellular cytokine staining; RNAseq, RNA sequencing.

SUPPLEMENTARY FIGURE 2 Pairwise correlations of day of challenge measurements among the 20 top-ranked immune response variables for the MAL068 full analysis (RRR and ARR) (i.e. the variables shown in Figure 3A). Cell color indicates the correlation between variables (scale bar at upper right of plot). The two columns on the left indicate the odds ratio for post-challenge protection against patent parasitemia per unit-increase in a given immune response variable on challenge day (“Odds Ratio”) and assay type (“Assay”). ADCP, antibody-dependent cellular phagocytosis; ADNKA, antibody-dependent natural killer cell activation; BAMA, binding antibody multiplex assay; BLI, biolayer interferometry; ELISA, enzyme-linked immunosorbent assay.

SUPPLEMENTARY FIGURE 3 Pairwise correlations of day of challenge measurements among the 338 down selected immune response variables for the MAL071 full analysis (RRR and RRr) (i.e. the variables listed in Table S1). Cell color indicates the correlation between variables (scale bar at upper right of plot). The two columns on the left indicate the odds ratio for post-challenge protection against patent parasitemia per unit-increase in a given immune response variable on challenge day (“Odds Ratio”) and assay type (“Assay”). ADCC, antibody-dependent cellular cytotoxicity; ADCD, antibody-dependent complement deposition; ADCP, antibody-dependent cellular phagocytosis; ADDCP, antibody dependent dendritic cell phagocytosis; ADNKA, antibody-dependent natural killer cell activation; ADNP, antibody-dependent neutrophil phagocytosis; BAMA, binding antibody multiplex assay; BLI, biolayer interferometry; ELISA, enzyme-linked immunosorbent assay; ELISpot, enzym.

SUPPLEMENTARY FIGURE 4 Pairwise correlations of day of challenge measurements among the 20 top-ranked immune response variables for the MAL071 full analysis (RRR and RRr) (i.e. the variables shown in Figure 3C). Cell color indicates the correlation between variables (scale bar at upper right of plot). The two columns on the left indicate the odds ratio for post-challenge protection against patent parasitemia per unit-increase in a given immune response variable on challenge day (“Odds Ratio”) and assay type (“Assay”). ICS, intracellular cytokine staining; RNAseq, RNA sequencing.

SUPPLEMENTARY FIGURE 5 Cross-study validation (MAL071 RRr) of univariate and bivariate models. A, C) Forest plots of cross-validated AUC scores for the top-performing univariate and bivariate models trained on (A) the MAL068 RRR data set or (C) the entire MAL068 data set (RRR + ARR). All data were day-of-challenge. Dots show the cross-validated AUC calculated using the cvAUC package in R; whiskers extend through 95% empirical intervals of the 200 individual 5-fold cross-validation runs. B, D) Validation AUCs of the same models as evaluated in predicting post-challenge outcomes in the RRr arm of MAL071. Purple, single-variable model; orange, two-variable model. The slightly enlarged bubbles in (B) and (D) identify the top-performing models as assessed by CV-AUC (A, C).

SUPPLEMENTARY FIGURE 6 Trained CV-AUC on MAL068 (all data or RRR) versus validation AUC on MAL071 (all, RRr, and RRR) for the top 200 uni, bi-, and tri-variate models.

SUPPLEMENTARY FIGURE 7 Heterogeneity across participants in ability of models to correctly predict challenge outcome. Using the top 20 best-performing univariate models (as assessed by CV-AUC) in predicting challenge outcome in the MAL068 RRR arm, leave-one-out prediction was done for MAL068 RRR (bottom box) and prediction was done for MAL071 RRR (top box) (models trained on MAL068 RRR). In the boxplots below, each dot corresponds to a trial participant and the color of the dot (pink: infected; blue: protected) displays that individual’s actual challenge outcome. The dots are plotted according to the proportion of models that correctly predict challenge outcome for each individual and stratified by trial.

SUPPLEMENTARY TABLE 1 Lists of variables that passed immunogenicity down-selection for the day-of-challenge MAL068 data set (RRR and ARR) and for the day-of-challenge MAL071 data set (RRR and RRr).

SUPPLEMENTARY TABLE 2 Top 20 most predictive variables in univariate models predicting challenge outcomes in MAL068 (RRR and ARR combined) or in MAL071 (RRR and RRr combined).

SUPPLEMENTARY TABLE 3 Top 20 variables of each PC for MAL068 (RRR and ARR combined) and MAL071 (RRR and RRr combined).

SUPPLEMENTARY TABLE 4 Variables, CV-AUCs, validation AUCs, and direction of association with protection (protection or non-protection) for the top 10 univariate and top 10 bivariate models assessed in cross-study validation. Models were trained on either MAL068 RRR or all MAL068 data (RRR + ARR) and validated on MAL071 RRR data.

## Abbreviations

CEVAC, Center for Vaccinology (Ghent University, Ghent, Belgium); CIDR, Center for Infectious Disease Research (Seattle, WA, United States); GSK, GlaxoSmithKline; MGH, Massachusetts General Hospital; WRAIR, Walter Reed Army Institute of Research (Silver Spring, MD, United States).
