# Supplementary material for: Comprehensive Data Integration Approach to Assess Immune Responses and Correlates of RTS,S/AS01-Mediated Protection From Malaria Infection in Controlled Human Malaria Infection Trials
Source: Front Big Data. 2021 Jun 15;4:672460. doi: 10.3389/fdata.2021.672460 (PMC8239149; doi:10.3389/fdata.2021.672460)

Supplementary Material

1 Supplementary Tables

Supplementary Table 1. Lists of variables that passed immunogenicity down-selection for the day-of-challenge MAL068 data set (RRR and ARR) and for the day-of-challenge MAL071 data set (RRR and RRr).

Supplementary Table 2. Top 20 most predictive variables in univariate models predicting challenge outcomes in MAL068 (RRR and ARR combined) or in MAL071 (RRR and RRr combined).

Supplementary Table 3. Top 20 variables of each PC for MAL068 (RRR and ARR combined) and MAL071 (RRR and RRr combined).

Supplementary Table 4. Variables, CV-AUCs, validation AUCs, and direction of association with protection (protection or non-protection) for the top 10 univariate and top 10 bivariate models assessed in cross-study validation. Models were trained on either MAL068 RRR or all MAL068 data (RRR + ARR) and validated on MAL071 RRR data.

2 Supplementary Figures

Supplementary Figure 1. Pairwise correlations of day of challenge measurements among the 275 down selected immune response variables for the MAL068 full analysis (RRR and ARR) (i.e. the variables listed in Table S1). Cell color indicates the correlation between variables (scale bar at upper right of plot). The two columns on the left indicate the odds ratio for post-challenge protection against patent parasitemia per unit-increase in a given immune response variable on challenge day ("Odds Ratio") and assay type ("Assay"). ADCC, antibody-dependent cellular cytotoxicity; ADCD, antibody-dependent complement deposition; ADCP, antibody-dependent cellular phagocytosis; ADDCP, antibody dependent dendritic cell phagocytosis; ADNKA, antibody-dependent natural killer cell activation; ADNP, antibody-dependent neutrophil phagocytosis; BAMA, binding antibody multiplex assay; BLI, biolayer interferometry; ELISA, enzyme-linked immunosorbent assay; ELISpot, enzyme-linked immunospot; ICS, intracellular cytokine staining; RNAseq, RNA sequencing.

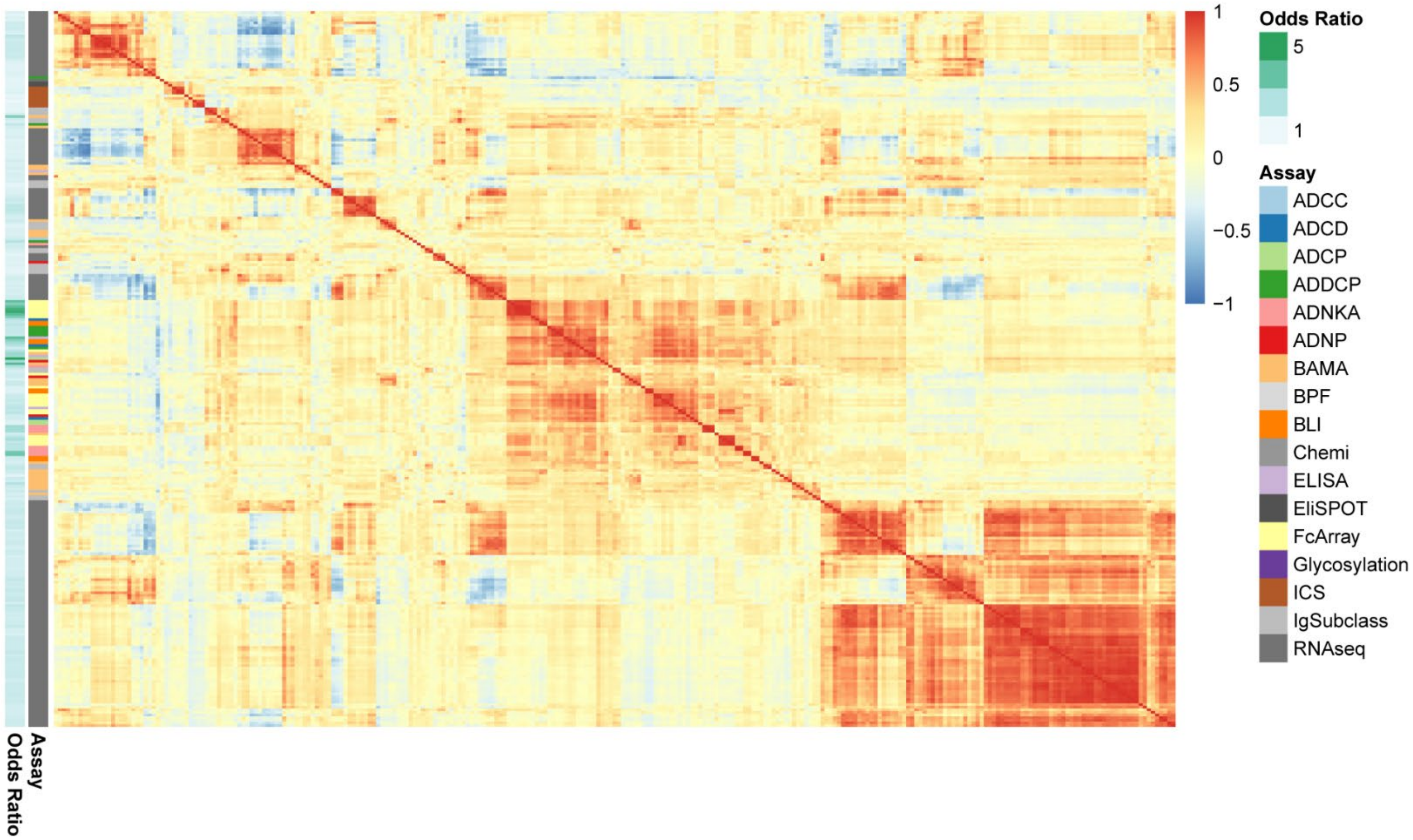

Supplementary Figure 2. Pairwise correlations of day of challenge measurements among the 20 top-ranked immune response variables for the MAL068 full analysis (RRR and ARR) (i.e. the variables shown in Figure 3A). Cell color indicates the correlation between variables (scale bar at upper right of plot). The two columns on the left indicate the odds ratio for post-challenge protection against patent parasitemia per unit-increase in a given immune response variable on challenge day ("Odds Ratio") and assay type ("Assay"). ADCP, antibody-dependent cellular phagocytosis; ADNKA, antibody-dependent natural killer cell activation; BAMA, binding antibody multiplex assay; BLI, biolayer interferometry; ELISA, enzyme-linked immunosorbent assay.

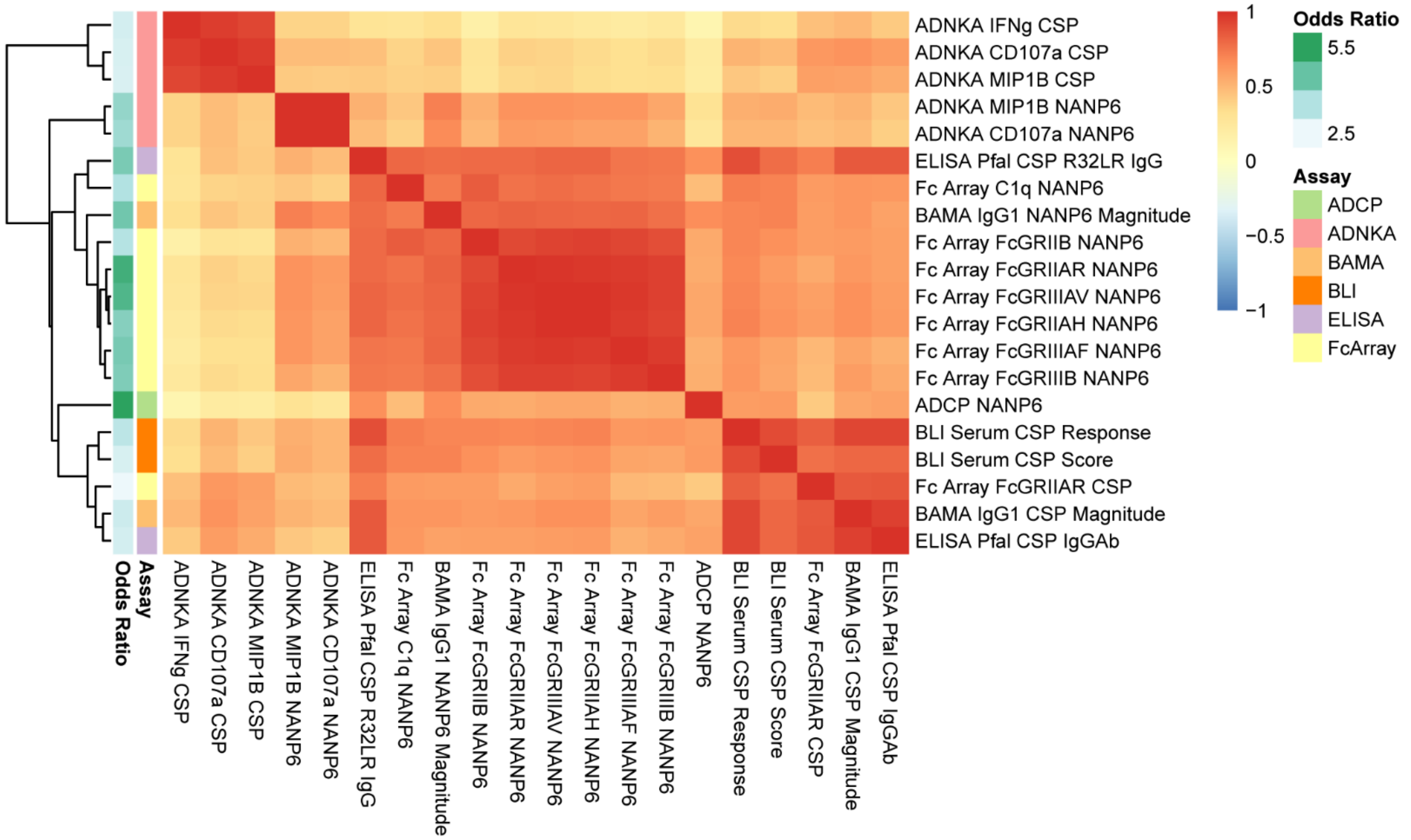

Supplementary Figure 3. Pairwise correlations of day of challenge measurements among the 338 down selected immune response variables for the MAL071 full analysis (RRR and RRr) (i.e. the variables listed in Table S1). Cell color indicates the correlation between variables (scale bar at upper right of plot). The two columns on the left indicate the odds ratio for post-challenge protection against patent parasitemia per unit-increase in a given immune response variable on challenge day ("Odds Ratio") and assay type ("Assay"). ADCC, antibody-dependent cellular cytotoxicity; ADCD, antibody-dependent complement deposition; ADCP, antibody-dependent cellular phagocytosis; ADDCP, antibody dependent dendritic cell phagocytosis; ADNKA, antibody-dependent natural killer cell activation; ADNP, antibody-dependent neutrophil phagocytosis; BAMA, binding antibody multiplex assay; BLI, biolayer interferometry; ELISA, enzyme-linked immunosorbent assay; ELISpot, enzyme-linked immunospot; ICS, intracellular cytokine staining; RNAseq, RNA sequencing.

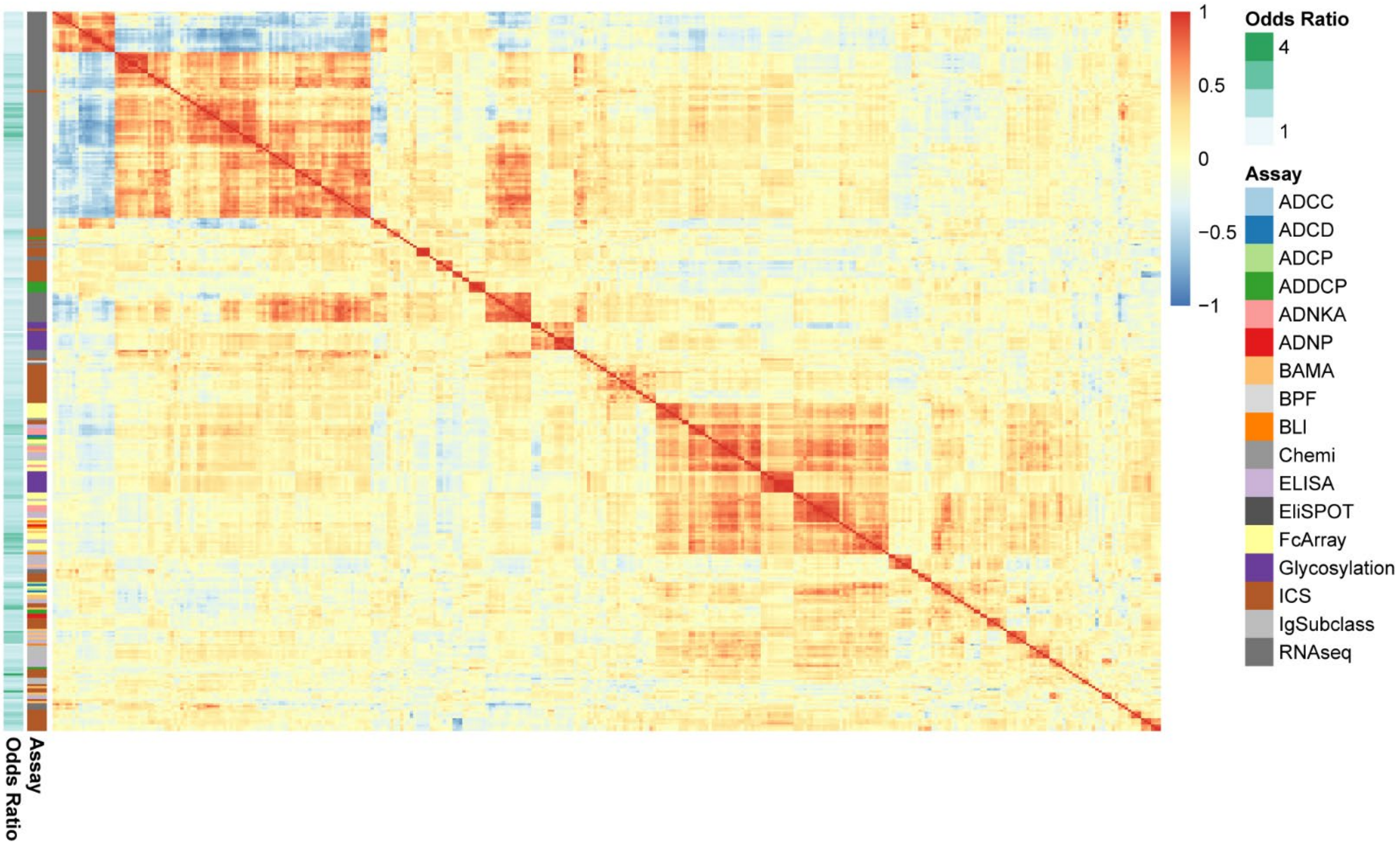

Supplementary Figure 4. Pairwise correlations of day of challenge measurements among the 20 top-ranked immune response variables for the MAL071 full analysis (RRR and RRr) (i.e. the variables shown in Figure 3C). Cell color indicates the correlation between variables (scale bar at upper right of plot). The two columns on the left indicate the odds ratio for post-challenge protection against patent parasitemia per unit-increase in a given immune response variable on challenge day ("Odds Ratio") and assay type ("Assay"). ICS, intracellular cytokine staining; RNAseq, RNA sequencing.

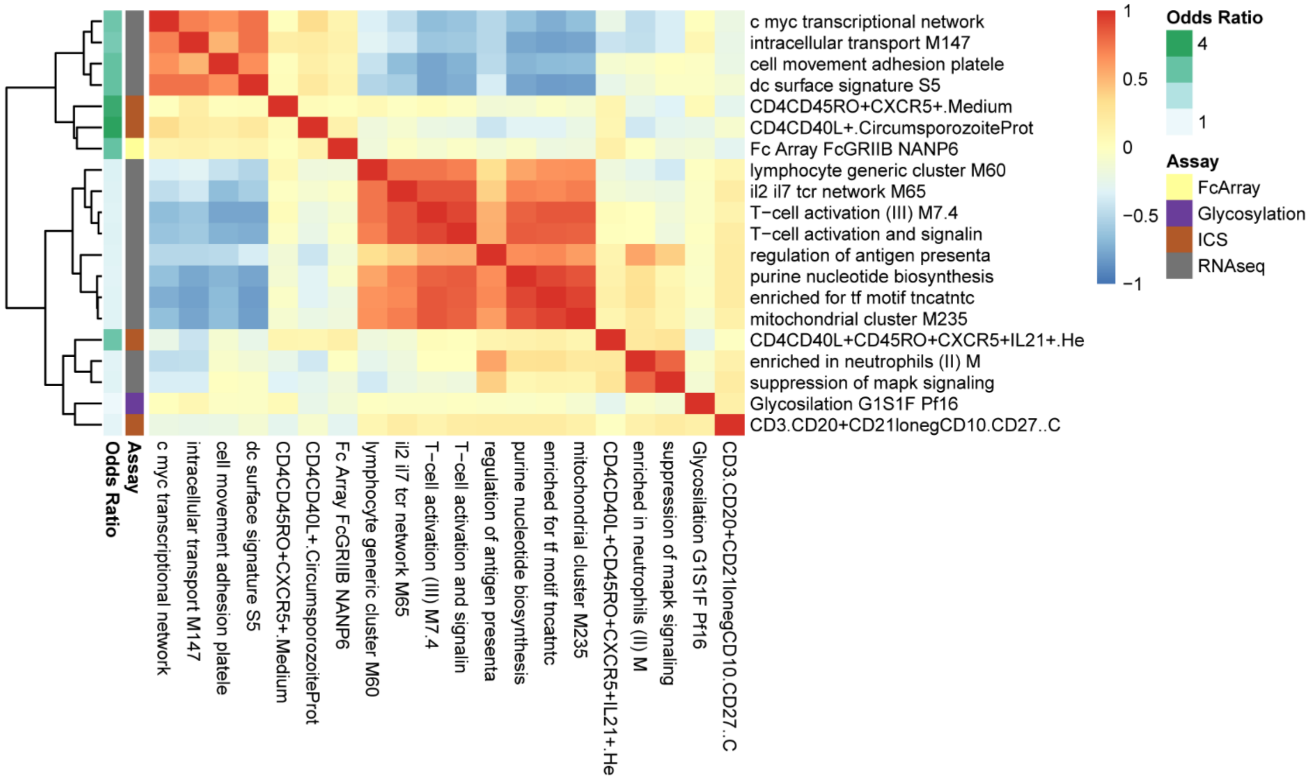

Supplementary Figure 5. Cross-study validation (MAL071 RRr) of univariate and bivariate models. A, C) Forest plots of cross-validated AUC scores for the top-performing univariate and bivariate models trained on (A) the MAL068 RRR data set or (C) the entire MAL068 data set (RRR + ARR). All data were day-of-challenge. Dots show the cross-validated AUC calculated using the cvAUC package in R; whiskers extend through 95% empirical intervals of the 200 individual 5-fold cross-validation runs. B, D) Validation AUCs of the same models as evaluated in predicting post-challenge outcomes in the RRr arm of MAL071. Purple, single-variable model; orange, two-variable model. The slightly enlarged bubbles in (B) and (D) identify the top-performing models as assessed by CV-AUC (A, C).

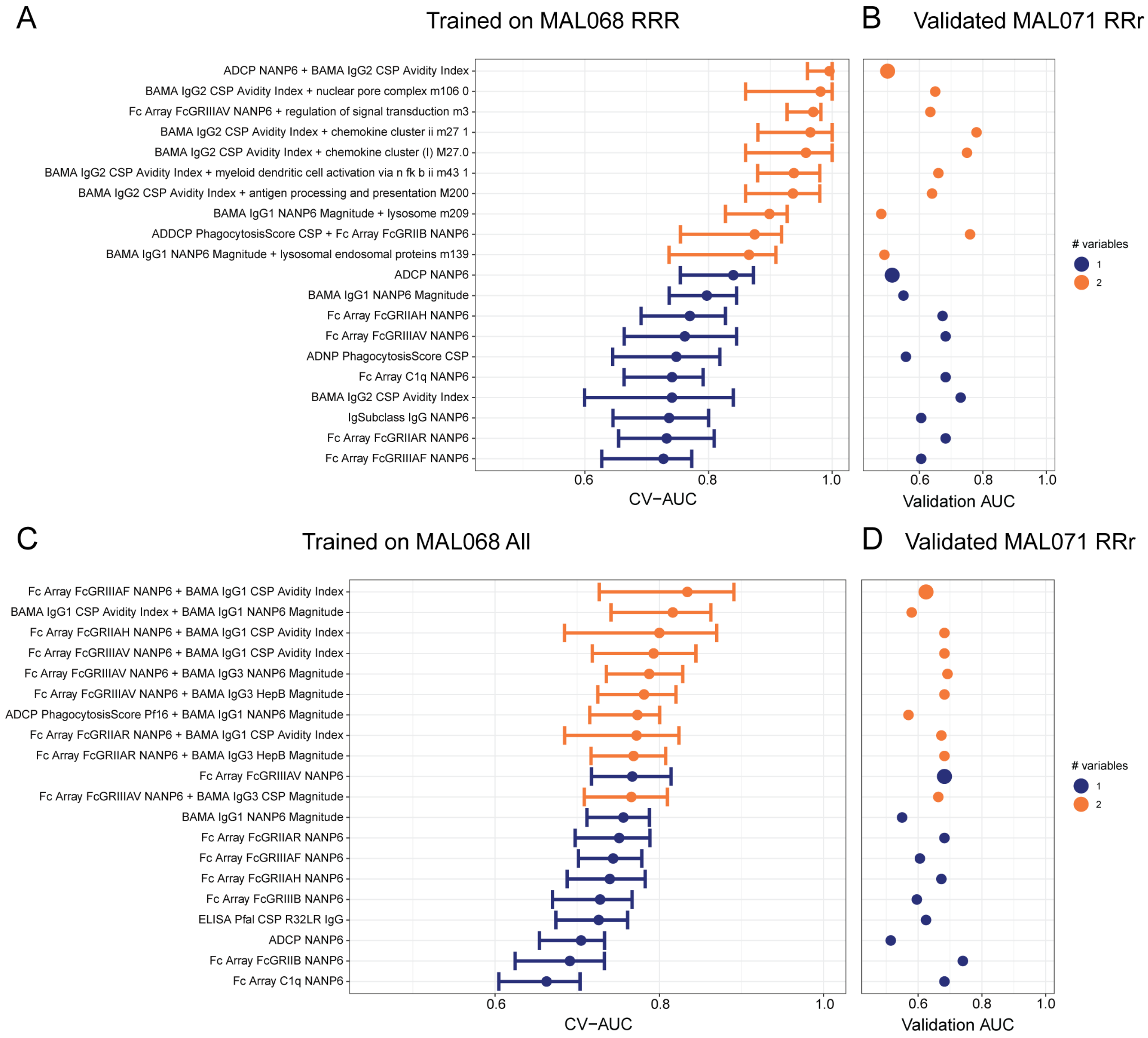

Supplementary Figure 6. Trained CV-AUC on MAL068 (all data or RRR) versus validation AUC on MAL071 (all, RRr, and RRR) for the top 200 uni, bi-, and tri-variate models.

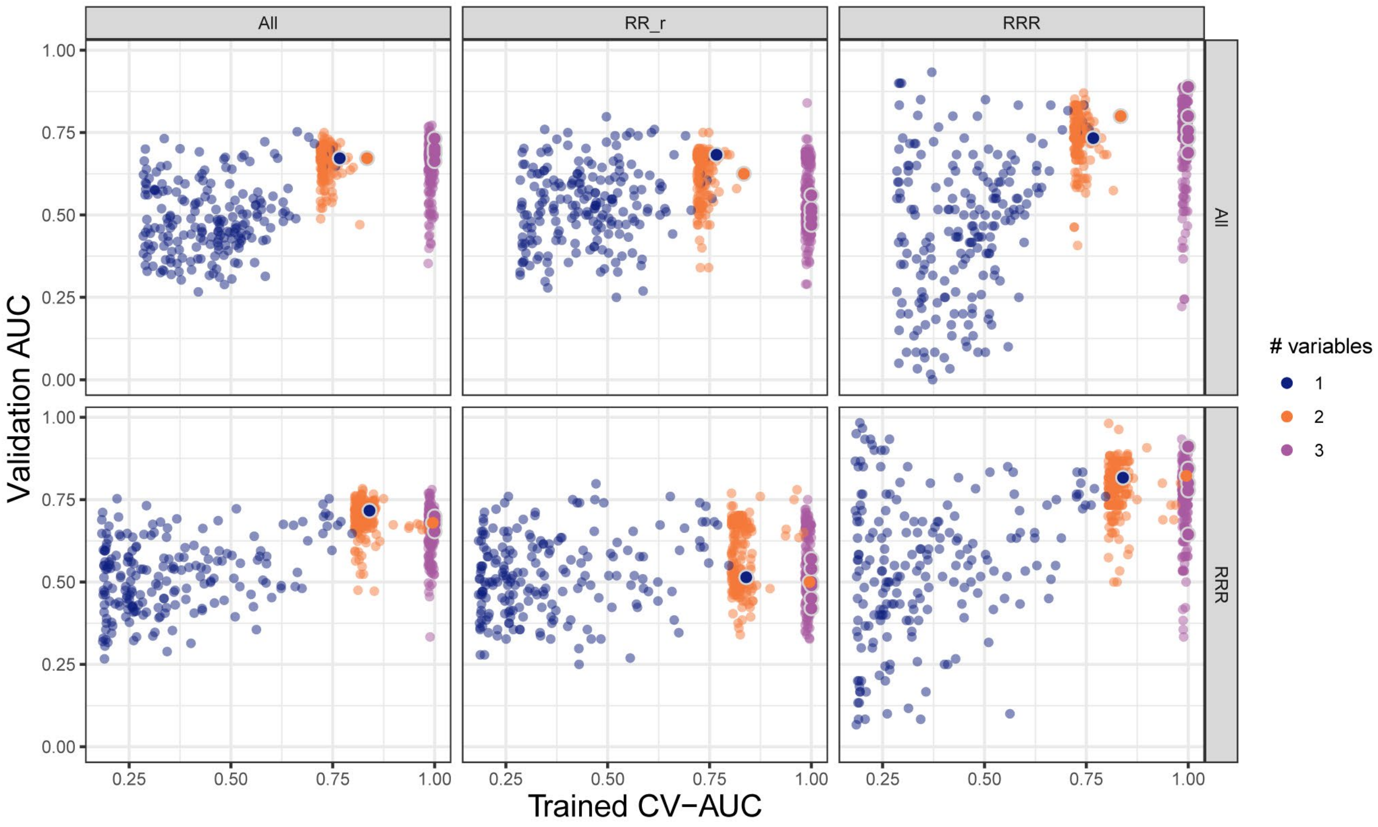

Supplementary Figure 7. Heterogeneity across participants in ability of models to correctly predict challenge outcome. Using the top 20 best-performing univariate models (as assessed by CV-AUC) in predicting challenge outcome in the MAL068 RRR arm, leave-one-out prediction was done for MAL068 RRR (bottom box) and prediction was done for MAL071 RRR (top box) (models trained on MAL068 RRR). In the boxplots below, each dot corresponds to a trial participant and the color of the dot (pink: infected; blue: protected) displays that individual's actual challenge outcome. The dots are plotted according to the proportion of models that correctly predict challenge outcome for each individual and stratified by trial.

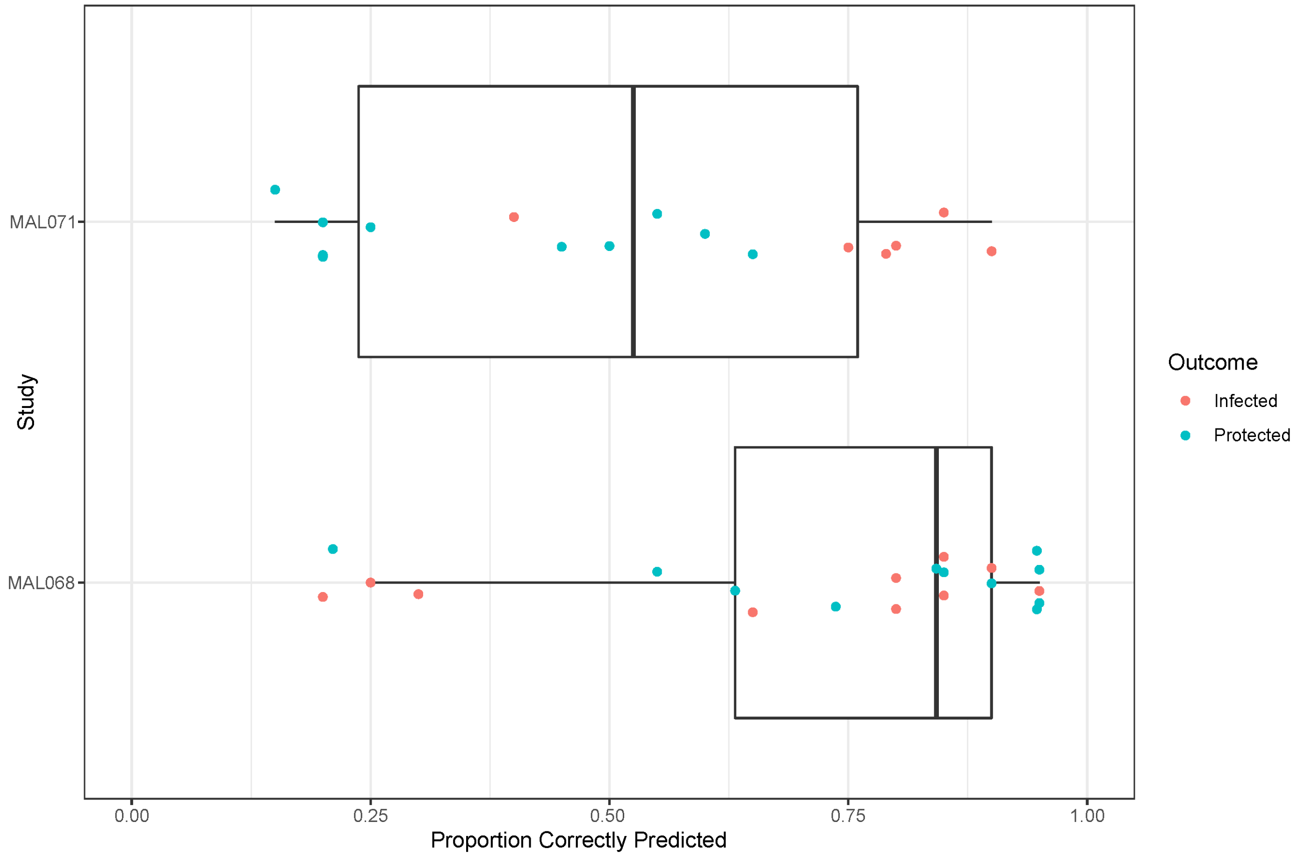

Supplement: Supplementary file 5 [file Presentation1.PDF]
